# Natural bioactive molecules and chemotherapeutics synergism for enhanced cancer therapy

**DOI:** 10.1186/s12986-026-01094-4

**Published:** 2026-02-21

**Authors:** Mehrdad Hashemi, Katayoun Heshmatzad, Ghazaleh Shahsavan, Vahid Tavakolpour, Sara Komeilie Esfahani, Pardis Karimi, Naghmeh Beikzadeh, Saba Mashhadikhan, Sevda Nasirzade, Ali Vasheghani Farahani, Neda Zali, William C. Cho, Afshin Taheriazam, Ehsan Maghrebi-Ghojogh, Mina Alimohammadi, Payman Rahimzadeh, Kiavash Hushmandi, Maliheh Entezari

**Affiliations:** 1 Farhikhtegan Medical Convergent Sciences Research Center, Farhikhtegan Hospital, Faculty of Medicine, TeMs.C., Islamic Azad University, Tehran, Iran; 2 Department of Genetics, Faculty of Advanced Science and Technology, TeMs.C., Islamic Azad University, Tehran, Iran; 3 Department of Biochemistry and Molecular Biology, Cumming School of Medicine, University of Calgary, Calgary, AB Canada; 4 Department of Stem Cells and Regenerative Medicine, Institute of Medical Biotechnology, National Institute of Genetic Engineering and Biotechnology (NIGEB), Tehran, Iran; 5 Basic and Molecular Epidemiology of Gastrointestinal Disorders Research Center, Research Institute for Gastroenterology and Liver Diseases, Shahid Beheshti University of Medical Sciences, Tehran, Iran; 6 Department of Clinical Oncology, Queen Elizabeth Hospital, Kowloon, Hong Kong China; 7 Department of Orthopedics, Faculty of Medicine, TeMs.C., Islamic Azad University, Tehran, Iran; 8 Pharmaceutical Sciences Research Center, Faculty of Pharmacy, Mazandaran University of Medical Sciences, Sari, Iran; 9 Department of Immunology, School of Medicine, Shahid Beheshti University of Medical Sciences, Tehran, Iran; 10 Surgical Research Society (SRS), Students’ Scientific Research Center, Tehran University of Medical Sciences, Tehran, Iran; 11 Department of Epidemiology, University of Tehran, Tehran, Iran

**Keywords:** Natural bioactives, Chemotherapy resistance, Synergistic cancer therapy, Phytochemicals

## Abstract

**Background:**

Chemotherapy remains a foundation of cancer care but is limited by multidrug resistance, systemic toxicities, and suboptimal selectivity, prompting interest in adjunctive strategies that improve efficacy and tolerability without adding significant burden to patients or healthcare systems.

**Aims/Objectives:**

This review highlights evidence on natural bioactive compounds, including polyphenols, alkaloids, terpenoids, and fungal metabolites, as adjuvants to standard chemotherapeutics, with objectives to: first, delineate mechanisms by which these agents enhance cytotoxic efficacy and overcome resistance; second, summarize preclinical and clinical combination data; and third, evaluate their potential to mitigate chemotherapy-induced organ toxicities through pathway modulation.

**Results:**

Natural bioactives modulate key oncogenic and stress-response pathways, such as NF-κB, PI3K/AKT/mTOR, and NRF2/HO-1, thereby sensitizing tumors to chemotherapy, attenuating pro-survival signaling, and enhancing apoptosis while reducing inflammatory and oxidative injury in normal tissues. Exemplary combinations, including curcumin with 5‑fluorouracil and resveratrol with cisplatin, have demonstrated improved antitumor activity and reduced toxicity in preclinical models, with early clinical observations supporting feasibility and safety in selected settings. Additionally, several compounds exhibit organ-protective effects against cardiotoxicity, nephrotoxicity, neurotoxicity, and gastrointestinal injury induced by chemotherapy, suggesting dual benefits on efficacy and tolerability profiles.

**Conclusions:**

Integrating natural bioactives with conventional chemotherapy represents a promising strategy to enhance therapeutic index by concurrently amplifying antitumor mechanisms and mitigating dose‑limiting toxicities, though broader clinical validation and standardized quality controls are needed for routine adoption.

**Graphical abstract:**

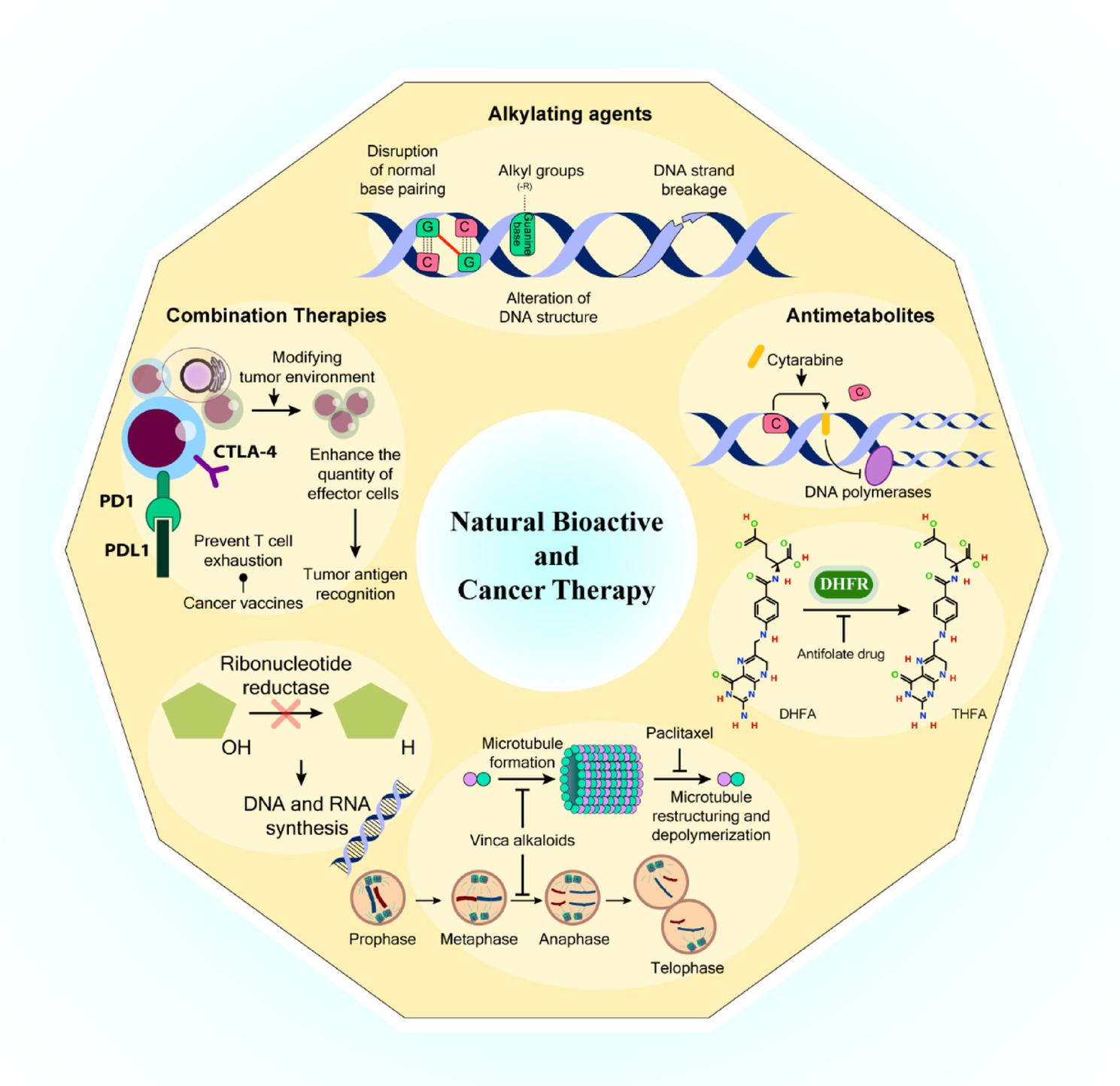

## Key points

•Cancer incidence and mortality rates are rising worldwide, posing significant public health challenges

•Chemotherapy remains the primary treatment despite tumor resistance and severe patient side effects.

•Combination therapies targeting multiple pathways improve efficacy and reduce harmful chemotherapy side effects.

## Introduction

Cancer represents one of the most significant threats to human life globally, with both occurrence rates and death rates showing an upward trend, presenting a major challenge to public health [1]. While multiple treatment approaches exist, including surgical intervention, chemotherapeutic methods, radiotherapy, and immune-based treatments, chemotherapy remains the primary therapeutic strategy. Nevertheless, a growing concern has emerged as tumors develop increased resistance to chemical treatments over time, diminishing the effectiveness of these anti-cancer medications [2]. Furthermore, the severe side effects experienced by patients undergoing chemotherapy pose an additional critical challenge that requires immediate attention in drug development [2].

Understanding the intricate nature of cancer remains a significant scientific challenge in developing effective treatments that can address the limitations of conventional chemotherapy, including whole-body toxicity, poor target specificity, and drug resistance issues that compromise treatment outcomes. However, significant advances in understanding cancer development mechanisms over recent decades have illuminated key characteristics of cancerous cells during their initiation and progression, leading to innovative therapeutic approaches [3, 4].

The implementation of combination chemotherapy, which utilizes multiple anticancer drugs targeting different molecular pathways, has shown promise in enhancing treatment efficacy while reducing harmful side effects [5]. Additionally, there has been growing interest in exploring natural compounds as alternative treatments due to their lower toxicity profiles. Research has demonstrated that combining traditional anticancer medications with natural substances such as resveratrol and curcumin in colorectal cancer, and epigallocatechin-3-gallate (EGCG) in ovary cancer, shows potential in combating treatment resistance and providing protective effects against chemotherapy-induced damage [6–8].

Inflammation plays a vital role within the tumor microenvironment, significantly impacting cancer cell growth and tumor progression [9]. Research has demonstrated that inflammatory cytokines operate primarily through activating nuclear factor kappa-light-chain-enhancer of activated B cells (NF-κB), a crucial transcription factor. This factor triggers multiple genes that regulate cell death, malignant transformation, tumor growth, invasiveness, spread, survival, and resistance to both chemical and radiation treatments. It also influences inflammatory responses in both initial and advanced aggressive cancers. Consequently, researchers have identified various naturally occurring compounds with immune-modulating properties as potential chemotherapy supplements [10]. However, natural substances carry their own risks, necessitating careful evaluation of their possible adverse effects and interactions with both conventional cancer drugs and other natural compounds.

While chemotherapy remains the primary cancer treatment approach, the increasing resistance of cancer cells to various therapeutic agents, including both traditional and targeted medications, has become a prevalent issue. A significant proportion of cancer-related deaths can be linked to this drug resistance phenomenon [11]. Although developing strategies to combat treatment resistance presents a substantial challenge, certain natural substances, encompassing diverse chemical structures and therapeutic properties, have shown promise in counteracting cancer drug resistance. This review examines how natural compounds, including polyphenols, alkaloids, and terpenoids, can enhance therapeutic outcomes and help overcome chemotherapy resistance.

## Natural bioactive products

The search for natural substances with anticancer properties has ancient roots, tracing back to many years ago. However, systematic scientific research in this field only began in the 1950s, leading to the discovery of several plant-based anticancer compounds. These include derivatives of vinca alkaloids, camptothecin, podophyllotoxin, endophytic fungi-derived compounds, and semi-synthetic taxol analogs, all of which have become valuable therapeutic agents in cancer treatment [12, 13]. Between the 1960s and 1980s, the US National Cancer Institute (NCI) conducted extensive screening of potential anticancer compounds, examining over 180,000 microbial sources, 16,000 marine organisms, and 114,000 plant-derived substances [14]. The development of plant-based medications has also enabled researchers to create more effective and safer anti-tumor drugs by understanding the collaborative effects between various components found in anti-tumor herbs.

**Fig. 1:**
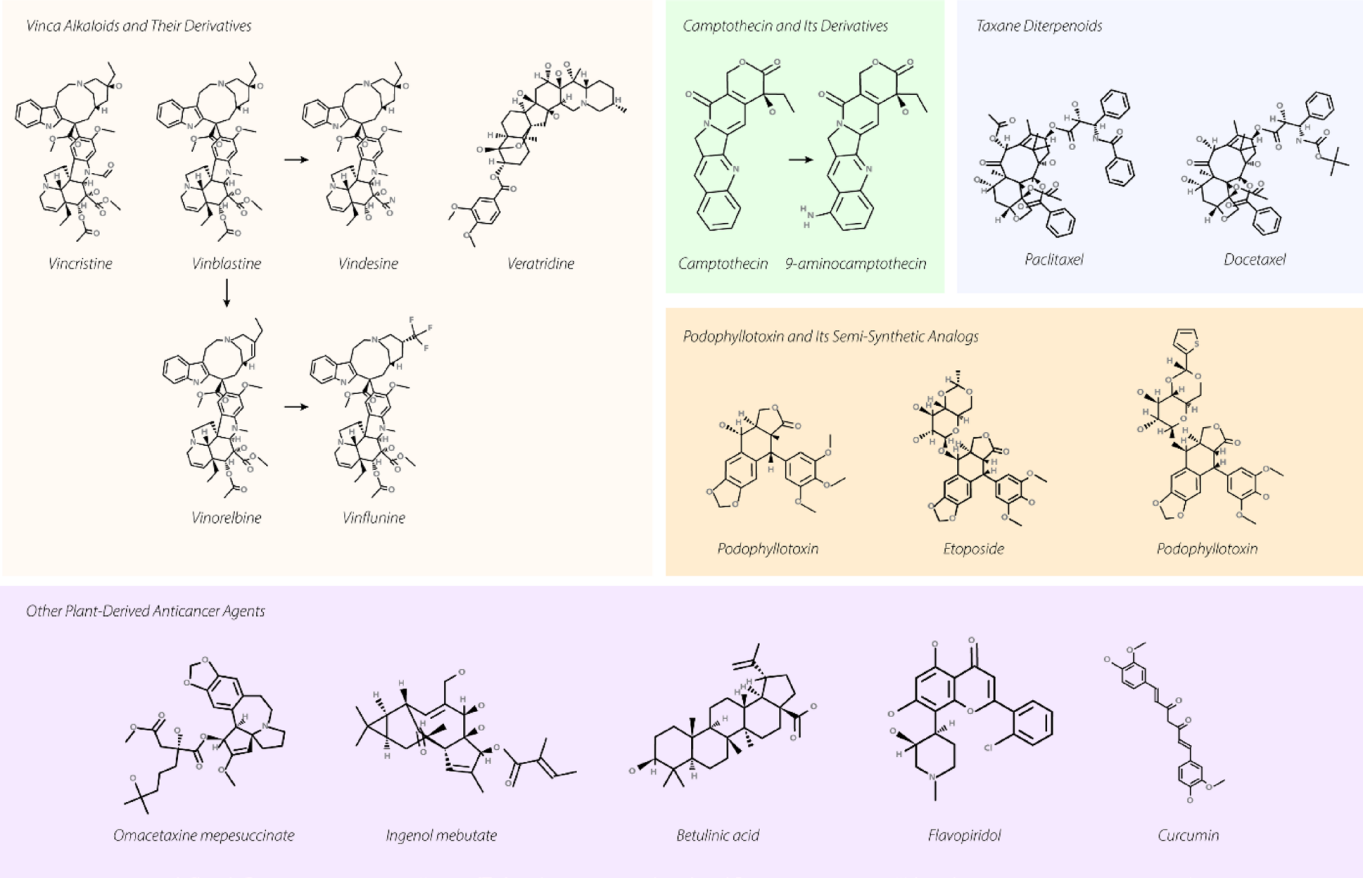
Natural Bioactive Compounds in Cancer Therapy. Vinca alkaloids, camptothecin, taxanes, podophyllotoxin, and their derivatives, together with some other plant-derived compounds, such as Omacetaxine, Curcumin, Flavopiridol, etc. are among the best-studied natural bioactive compounds in cancer therapy. The figure was created in Adobe Illustrator

Natural products represent a viable and effective resource for treating various conditions and life-threatening illnesses, including cancer (Fig. 1). Recent years have witnessed increased recognition of bioactive compounds and natural substances as sources of cancer-fighting medications within an integrated, multidisciplinary framework. The therapeutic properties of plants have been recognized throughout human history [15]. Currently, natural sources contribute to more than half of contemporary clinical medications, demonstrating significant anti-cancer properties [16].

The US National Cancer Institute (NCI) pioneered the systematic investigation of natural anticancer agents, leading to significant discoveries in natural tumor-fighting compounds [17]. The development of plant-derived medications requires specific cultivation conditions and careful resource management. For instance, in 1998, Sohn and colleagues reported that producing 1 kg of paclitaxel required processing 10,000 kg of yew tree bark, with 38,000 trees needed to generate 25 kg of paclitaxel for treating 12,000 cancer patients [18]. While plant collection for cancer drug discovery ceased in 1982, new screening methods implemented in 1986 enhanced plant research and organism collection, particularly in tropical and subtropical regions. Graham et al. in an extension of Hartwell’s comprehensive work declared over 3,000 plants with potential cancer-fighting properties [19]. Although various cancer medications are available, their application is often limited by toxic side effects [20]. The natural world’s biodiversity continues to serve as a crucial source of remarkable anti-cancer compounds [21–23].

### Plant-derived bioactives

Throughout the past decades, scientists have extensively studied the traditional medicinal uses and pharmacological properties of various plant-extracted bioactive substances, with recent focus expanding to include their effectiveness against microbes and biofilm formation. Numerous laboratory studies and animal experiments have demonstrated the medicinal value of diverse plant compounds. Among the most widely recognized plant-sourced cancer-fighting agents are the vinca alkaloids and their modified forms, camptothecin and its derivatives, modified versions of podophyllotoxin, and various terpene compounds.

#### Vinca alkaloids and their derivatives

The emergence of plants as anticancer agents began with the extraction of two key alkaloids - vincristine and vinblastine - from Catharanthus roseus (Madagascar periwinkle) [24]. These medications have been fundamental in cancer treatment for approximately five decades. Their therapeutic mechanism involves preventing tubulin molecules from polymerizing, which disrupts mitotic spindle formation, ultimately triggering cell death or stopping cells in metaphase [25]. The realm of plant-derived natural alkaloids used in cancer treatment includes various compounds such as vincristine, vinblastine, vinorelbine, vinflunine, veratridine, and berbamine.

Scientists have developed several modified versions of these two original alkaloid medications. Vindesine, created by substituting vinblastine’s C-acetyl group with an amino group, occasionally serves as a treatment option for a variety of cancers. Vinorelbine, another modified form of vinblastine, was developed by modifying the indole ring’s connection to piperidine nitrogen and removing water from the piperidine ring [26]. Similar to other modified vinca alkaloids, vinflunine works by binding to tubulin molecules, thereby preventing microtubule polymerization and causing tubulin para crystal formation [27, 28].

Research by Cao and colleagues examined the cancer-fighting capabilities of 13 different isoquinoline alkaloids derived from Hylomecon japonica against MCF-7 breast cancer cells. Seven of these compounds showed notable inhibitory effects with IC50 values below 20 µM: 6,10-dimethoxydihydrochelerythrine, 6 S/R-acroleinyldihydrochelerythrine, 9-methoxy-10-hydroxy-norchelerythrine, 10-methoxyboconoline, 6-methoxydihydrosanguinarine, dihydrosanguinarine, and 6-acetaldehydedihydrochelerythrine [29]. Separate studies by Freeling and team revealed the cancer-suppressing properties of veratridine (VTD), a plant alkaloid that enhances UBXN2A (an anti-tumor protein) expression by inhibiting mortalin, a protein known to promote cancer development [30].

Liu and colleagues demonstrated berbamine inhibits cell growth and migration in triple negative breast cancer cells. This alkaloid achieved its effects by modulating both the phosphatidylinositol-3 kinase (PI3K)/protein kinase B (Akt)/mechanistic (mammalian) target of rapamycin (mTOR) and PI3K/Akt/MDM2/p53 signaling pathways [31]. Further research by Esnaashari’s team explored the combined effects of doxorubicin and noscapine-loaded polymeric nanoparticles (NOS-NPs) in breast cancer treatment. Their experiments, conducted both in laboratory cell cultures (4T1 breast cancer cells) and living organisms (mice), showed that while doxorubicin and NOS-NPs alone achieved 32% and 55.10% growth inhibition respectively, their combination significantly increased inhibition to 68.50% [32].

#### Camptothecin and its derivatives

The plant Camptotheca acuminata contains camptothecin (CPT), a quinoline alkaloid with anticancer properties. CPT functions by blocking topoisomerase I, which leads to DNA disruption and eventual cell death [33]. However, clinical trials were discontinued due to CPT’s high toxicity levels and poor solubility in water.

Another modified version, 9-aminocamptothecin (9-AC), showed promise in laboratory tests but failed to demonstrate effective anticancer properties in clinical settings [34]. Later phase-II studies indicated that while the drug showed effectiveness against lymphoma and ovarian cancers, it proved ineffective in treating colon cancers. As a result, further development was halted in 1999 [35]. Additional clinical trials have investigated several related compounds, including: diflomotecan for treating advanced solid tumors (phase I) [36], gimatecan for advanced solid tumors (phase I) [37] and recurrent ovarian, peritoneal, or fallopian tumors (phase II) [38], elomotecan for advanced solid tumors (phase I) [39], and EZN-2208 for advanced malignancies [40].

#### Podophyllotoxin and its semi-synthetic analogs

The medicinal plant *Podophyllum peltatum* serves as a crucial source of podophyllotoxin, an anticancer substance. Two significant derivatives of this compound, teniposide and etoposide, have proven effective in treating various cancer types through their mechanism of blocking topoisomerase II enzyme activity [41, 42]. While these two derivatives help address certain challenges such as metabolic breakdown, limited water solubility, and drug resistance development, researchers have continued to develop more effective compounds. This pursuit of enhanced therapeutic effectiveness has led to the creation of various semi-synthetic versions. These compounds are either currently used as cancer medications or are undergoing clinical trials as potential new treatment options.

#### Taxane diterpenoids

The isolation of paclitaxel from Yew tree bark extract represents a milestone in natural product-based drug discovery [43]. This compound made history as the first discovered agent that stimulates microtubule formation, and it has proven effective in treating various cancers [44]. Scientists have since developed numerous derivatives, with docetaxel emerging as the first clinically utilized variant, demonstrating notable effectiveness against various tumor types [45, 46].

Despite their therapeutic success, both approved taxane medications - paclitaxel and docetaxel - face certain limitations. Scientists continue to work to minimize their adverse effects through structural modifications. These efforts have yielded new compounds with reduced toxic effects, better solubility, and improved cytotoxic properties. Research has shown that the P-glycoprotein efflux pump, highly expressed in the blood-brain barrier (BBB), limits the ability of both docetaxel and paclitaxel to penetrate this barrier [47–49].

In 2010, cabazitaxel, a newer taxane derivative, received FDA approval for use alongside prednisone in treating hormone-resistant and standard prostate cancers. This compound works by preventing cancer cell growth through tubulin stabilization and inhibition of microtubule breakdown [50]. Additionally, researchers are exploring nanoparticle-based delivery systems to enhance effectiveness. One example is Abraxane, a solvent-free, albumin-bound nanoparticle formulation of paclitaxel that functions as a mitotic inhibitor with significantly enhanced therapeutic effects. Ongoing research focuses on developing novel taxanes to improve therapeutic outcomes and pharmacological properties, potentially replacing current NSCLC treatments such as docetaxel and paclitaxel [51].

#### Other plant-derived anticancer agents

The FDA-approved anticancer compound omacetaxine mepesuccinate, derived from *Cephalotaxus harringtonia*, functions by disrupting protein translation. Specifically, it blocks protein elongation by interfering with the A-site and preventing proper aminoacyl tRNA positioning [52]. Another FDA and EMA-approved compound, ingenol mebutate, extracted from Euphorbia peplus sap, was sanctioned in 2012 as a gel treatment for acid keratosis. This compound combines angelic acid with a diterpene [53].

Betulinic acid, a pentacyclic triterpenoid naturally occurring in *Betula* species (Betulaceae family), was first isolated from *Ziziphus* species including M. oenoplia and *M. rugosa* [54]. This compound demonstrates other biological benefits, such as anti-gastric ulcer effects and anti-platelet activity properties [55, 56]. Research by Dai et al. demonstrated the anticancer potential of Taxus chinensis var. mairei (TC) against lung cancer through both laboratory and animal studies. Their findings showed that TC’s aqueous extract effectively fought cancer by degrading CD47 while maintaining low toxicity [57]. Similarly, Wu et al.‘s research on CPTC-2, a polysaccharide from the same plant variety, showed significant dose-dependent antitumor effects against gastric cancer cells (SGC-7901), as confirmed by flow cytometry and MTS assays [58].

Flavopiridol, a synthetic compound structurally similar to rohitukine from the Indian plant Dysoxylum binectariferum [59], targets cyclin-dependent kinases, including the cyclin T/CDK9 complex, inactivates p-TEFb and blocks most RNA polymerase II transcription, and also suppresses anti-apoptotic proteins and Mc1-1 regulation while affecting mitochondrial permeability [60–62]. It holds the distinction of being the first cyclin-dependent kinase inhibitor to reach clinical trials [63].

Curcumin, a polyphenol derived from Curcuma longa (turmeric), exhibits various therapeutic properties including anti-inflammatory, pain-relieving, antiseptic, and antioxidant effects [64]. Among the curcuminoids present in turmeric (including bisdemethoxycurcumin and demethoxycurcumin), curcumin shows the most significant therapeutic potential. Its anticancer properties work through multiple mechanisms, affecting biological pathways involved in oncogene expression, mutagenesis, metastasis, apoptosis, and cell cycle regulation in a variety of cancers, such as head and neck squamous cell carcinoma [65].

### Fungal-derived bioactives

Medicinal mushrooms and fungi have deep historical roots in traditional healing practices. The development of therapeutic compounds derived from mushrooms continues to expand due to their effectiveness in human systems [66]. Several mushroom genera, including Trametes, Ganoderma, Auricularia, Tremella, and Flammulina, are recognized for their significant anti-tumor and immunomodulatory benefits [67]. Scientists are increasingly investigating novel mushroom species and their metabolites, such as Ganoderma, particularly focusing on their antioxidant and anticancer properties [68].

A notable study by Rutckeviski and colleagues examined how Agaricus bisporus extract β-(1→6)-d-glucan works together with doxorubicin to combat breast cancer cells (MDA-MB-231). Their research demonstrated that the combination of A. bisporus and doxorubicin worked synergistically to reduce tumor cell viability by 31%. Furthermore, when β-(1→6)-d-glucan treatment was combined with doxorubicin, it increased the susceptibility of MDA-MB-231 cells to doxorubicin’s effects [69].

Research by Yoon and colleagues demonstrated that adenosine derivatives from *Cordyceps militaris* triggered autophagic cell death in ovarian cancer through the ENT1/AMPK/mTOR pathway [70]. Similarly, Ganoderma lucidum’s anticancer properties against ovarian cancer were studied by Cen et al., who found it activated the ERK pathway through reactive species induction [71]. Another study by Thimmaraju et al. examined HUP-2, a polysaccharide extracted from Hypsizygus ulmarius using hot water extraction, which showed significant inhibitory effects and cytotoxicity against prostate cancer cells (PC3) [72].

Fekry and colleagues investigated selenium-enriched *Pleurotus ostreatus* anticancer properties in colon cancer, finding it increased interleukin-6 (IL-6) and IL-10 production while reducing tumor necrosis factor - alpha (TNF-α) and targeting the RAF1 pathway [73]. Meng et al.‘s research on water-soluble polysaccharide from Boletus edulis demonstrated its ability to induce mitochondrial apoptosis and inhibit proliferation in breast cancer cells (Ca761, MDA-MB-231) [74]. Additionally, silver nanoparticles derived from Boletus edulis and Coriolus versicolor showed significant anticancer effects against colorectal, breast, and hepatocellular carcinoma cells through proliferation inhibition and ROS-generated apoptosis [75].

Agaricus blazei, a medicinally significant mushroom, demonstrates anti-tumor properties by increasing T-regulatory and plasmacytoid dendritic cells, while enhancing human leukocyte, immunoglobulin, and killer-immunoglobulin receptor gene levels [76]. Misgiati et al. extracted ergosterol from *Agaricus blazei* Murill using *n*-hexane and found it exhibited significant anticancer activity against MCF-7 cells, with an IC50 of 43.10 µg/mL, working through cell cycle inhibition and apoptosis induction [77]. Further research by Sun et al. isolated an RNA-protein complex (FA-2-b-B) from the same mushroom, which showed promising proapoptotic and antiproliferative effects against chronic myeloid leukemia, suggesting its potential as an alternative treatment approach [78].

A study by Jeitler and colleagues demonstrated that combining Agaricus sylvaticus with chemotherapy for 6 cycles led to a significant reduction in appetite loss. In contrast, patients receiving the placebo experienced various digestive issues including loss of appetite, nausea, vomiting, diarrhea, and constipation [79]. Another investigation focused on treating inoperable hepatocellular carcinoma patients with Coriolus versicolor. While the treatment group showed enhanced quality of life compared to the control group, no other significant differences were noted, leading researchers to recommend its use in supportive care [80].

In a separate study, researchers evaluated powdered Agaricus bisporus (white button mushroom) for treating prostate cancer patients. These patients typically show elevated prostate-specific antigen (PSA) levels, which can indicate disease recurrence. The findings revealed reduced myeloid-derived suppressor cells following treatment, with responsive patients showing higher baseline interleukin-15 levels than non-responsive ones [81]. Additionally, a phase-I clinical trial conducted by Torkelson and colleagues examined Trametes versicolor’s effects on immunocompromised breast cancer patients. Their findings revealed enhanced immune function, including increased natural killer cell activity, higher lymphocyte counts, and dose-related improvements in CD8 + T-cells and CD19 + B-cells. These results suggest Trametes versicolor as a potential immunotherapy option for immunocompromised breast cancer patients (Table 1) [82].

**Table 1:** Plant and Fungal-Derived bioactive compounds in cancer therapy

Bioactive compound	Source	Mechanism of action	Cancer type	References
Vinflunine	Semi-synthetic (vinorelbine analog)	Binds tubulin, inhibits microtubule polymerization, forms tubulin para crystals	Advanced bladder cancer	[83]
Berbamine	Plant-based alkaloid	Regulates PI3K/Akt/mTOR and PI3K/Akt/MDM2/p53 pathways	Breast cancer	[31]
Camptothecin (CPT)	*Camptotheca acuminata*	Inhibits topoisomerase-I, causes DNA damage, induces apoptosis	Various cancers	[33]
Irinotecan Topotecan	Semi-synthetic (CPT derivatives)	Inhibits topoisomerase-I, disrupts DNA replication and transcription	Colorectal cancer Breast cancer	[84, 85]
Paclitaxel	Yew tree bark	Promotes microtubule synthesis, arrests cell cycle	Breast cancer	[86]
Docetaxel	Semi-synthetic (taxane derivative)	Stabilizes tubulin, inhibits microtubule depolymerization	Prostate, breast cancer	[45]
Cabazitaxel	Semi-synthetic (taxane derivative)	Stabilizes tubulin, inhibits microtubule depolymerization	Metastatic castration-resistant prostate cancer	[50]
Omacetaxine	*Cephalotaxus harringtonia*	Inhibits protein translation elongation	Various cancers	[52]
Betulinic acid	*Betula* species	Anti-inflammatory, anti-tumor, anti-HIV properties	Various cancers	[54]
Flavopiridol	Synthetic (rohitukine analog)	Inhibits cyclin-dependent kinases (CDKs), induces apoptosis	Various cancers	[60, 61, 63]
Curcumin	*Curcuma longa* (turmeric)	Modulates oncogene expression, apoptosis, cell cycle regulation	Various cancers	[65]
Agaricus bisporus β-glucan	*Agaricus bisporus* mushroom	Synergizes with doxorubicin, enhances tumor cell sensitivity	Breast cancer (MDA-MB-231)	[69]
Cordyceps militaris adenosine	*Cordyceps militaris* mushroom	Induces autophagic death via ENT1/AMPK/mTOR pathway	Ovarian cancer	[70]
Ganoderma lucidum	*Ganoderma lucidum* mushroom	Activates ERK pathway via ROS	Ovarian cancer	[71]
Agaricus blazei ergosterol	*Agaricus blazei* mushroom	Inhibits cell cycle, induces apoptosis	Breast cancer (MCF-7)	[77]
Trametes versicolor	*Trametes versicolor* mushroom	Enhances immune response (NK cells, CD8 + T-cells, CD19 + B-cells)	Breast cancer (immune-compromised)	[82]

## Chemotherapeutic drugs

The concept of cancer was first documented by Hippocrates, who is renowned as medicine’s founding father. He introduced the term ‘carcinos’ based on the crab-like appearance of cancerous growths. Later, this Greek term was translated to the Latin word ‘cancer’ by Celsus. Historical documents reveal that various civilizations identified different types of cancers and developed diverse therapeutic approaches [87, 88]. However, treatment methodologies remained largely unchanged until the 1900s [89].

Greek medical practitioners utilized *Colchicum autumnale* plant extracts for tumor reduction. Notably, in the 1930s, researchers discovered that colchicine, the extract’s active component, could disrupt microtubule formation, suggesting its potential therapeutic applications [90]. Similar anti-cancer properties were later recognized in compounds like vincristine and vinblastine [91].

The emergence of modern oncology coincided with World War II, when researchers began identifying active compounds following detailed genetic and molecular disease characterization [92]. Subsequently, chemotherapy was developed for treating cancers, accompanied by supportive care measures to improve patient outcomes [93].

During the latter half of the 1900s, researchers discovered various cancer triggers, including genetic mutations, environmental factors (such as toxic substances and viruses), and metabolic changes [3].

Cancer treatment poses significant challenges due to the metabolic similarities between cancer cells and normal cells [94, 95]. Drug development prioritizes selectivity - aiming to eliminate cancer cells while preserving healthy ones. While this approach shares similarities with antimicrobial therapy, cancer cells and bacteria differ substantially in their metabolic and physiological characteristics [96, 97]. Unlike bacteria, which are easily identified by immune cells, cancer cells can mask their surface markers to avoid immune detection .

Early cancer detection significantly improves treatment outcomes and survival rates. However, identifying transformed cells remains a major challenge in oncology research because early-stage biomarkers are often undetectable, and cancer cells’ ability to evade immune recognition suppresses natural immune responses [98].

Within tumor populations, transformed cells exhibit significant variations in physiological characteristics, metabolic processes, and growth rates [99, 100]. The genetic diversity among cancer cells within a tumor mass creates additional treatment challenges due to tumor heterogeneity [101]. To address these complexities, physicians employ combination drug therapies, tailoring treatment regimens to specific cancer types and stages [102].

### Commonly used chemotherapeutic drugs

Alkylating agents represent a primary category of contemporary cancer medications that function by disrupting DNA double-strand formation. These compounds operate by transferring alkyl groups to DNA’s guanine bases. The therapeutic effect occurs through multiple mechanisms, including DNA-protein cross-linking, alteration of DNA structure, disruption of normal base pairing, and DNA strand breakage. These changes ultimately lead to cell cycle arrest and permanent cellular senescence [103]. The mechanism involves electrophilic forms of these agents creating covalent bonds with cellular DNA, making them widely applicable across different cancer types. While alkylating agents are commonly prescribed as first-line treatments for various cancers, they demonstrate particularly high therapeutic efficacy against slow-progressing malignancies.

Antimetabolites form another crucial class of therapeutic agents that interfere with cellular metabolism by competing with, substituting for, or inhibiting specific cellular metabolites. These compounds typically mimic the structure of natural cellular metabolites or enzyme substrates that cells normally process for their metabolic requirements [104].

A key example involves the formation of tetrahydrofolate from dietary folate through dihydrofolate reductase. Several drugs, including aminopterin, methotrexate (amethopterin), pyrimethamine, trimethoprim, and triamterene, target this metabolic pathway by disrupting folate production [105]. Among these, methotrexate stands out as a particularly effective anticancer medication. It functions as an antifolate drug by inhibiting dihydrofolate reductase (DHFR), thereby preventing the conversion of dihydrofolic acid (DHFA) to tetrahydrofolic acid (THFA) [106]. This interference with coenzyme activity is crucial since these compounds play vital roles in nucleotide synthesis pathways. Due to its anti-inflammatory properties, methotrexate’s therapeutic applications extend beyond cancer treatment to include inflammatory conditions such as rheumatoid arthritis and severe psoriasis.

Pyrimidine-derived antimetabolites represent a class of cancer-fighting agents that achieve their cytotoxic effects by disrupting DNA synthesis. A notable example is cytarabine (also known as cytosine arabinoside or cytarabine), which interferes with both DNA and RNA synthesis by replacing cytosine with its arabinose-based variant. This drug is commonly used in treating acute myeloid leukemia [107]. Other significant members of this class include fluorouracil, 5-fluorouracil (5-FU), capecitabine, floxuridine, gemcitabine, decitabine, raltitrexed, and tegafur [108–113]. These compounds, structurally similar to purines or pyrimidines but with modified chemical groups, trigger cell death during S phase. They function either by incorrect incorporation into RNA and DNA or by blocking crucial enzymes involved in nucleic acid synthesis, particularly DNA polymerases, ribonucleotide reductase, and thymidylate synthetase. 5-FU has proven especially effective as a pyrimidine analog, significantly disrupting DNA and RNA synthesis, with its DNA incorporation leading to mitotic inhibition and cell death in dividing cells.

**Fig. 2:**
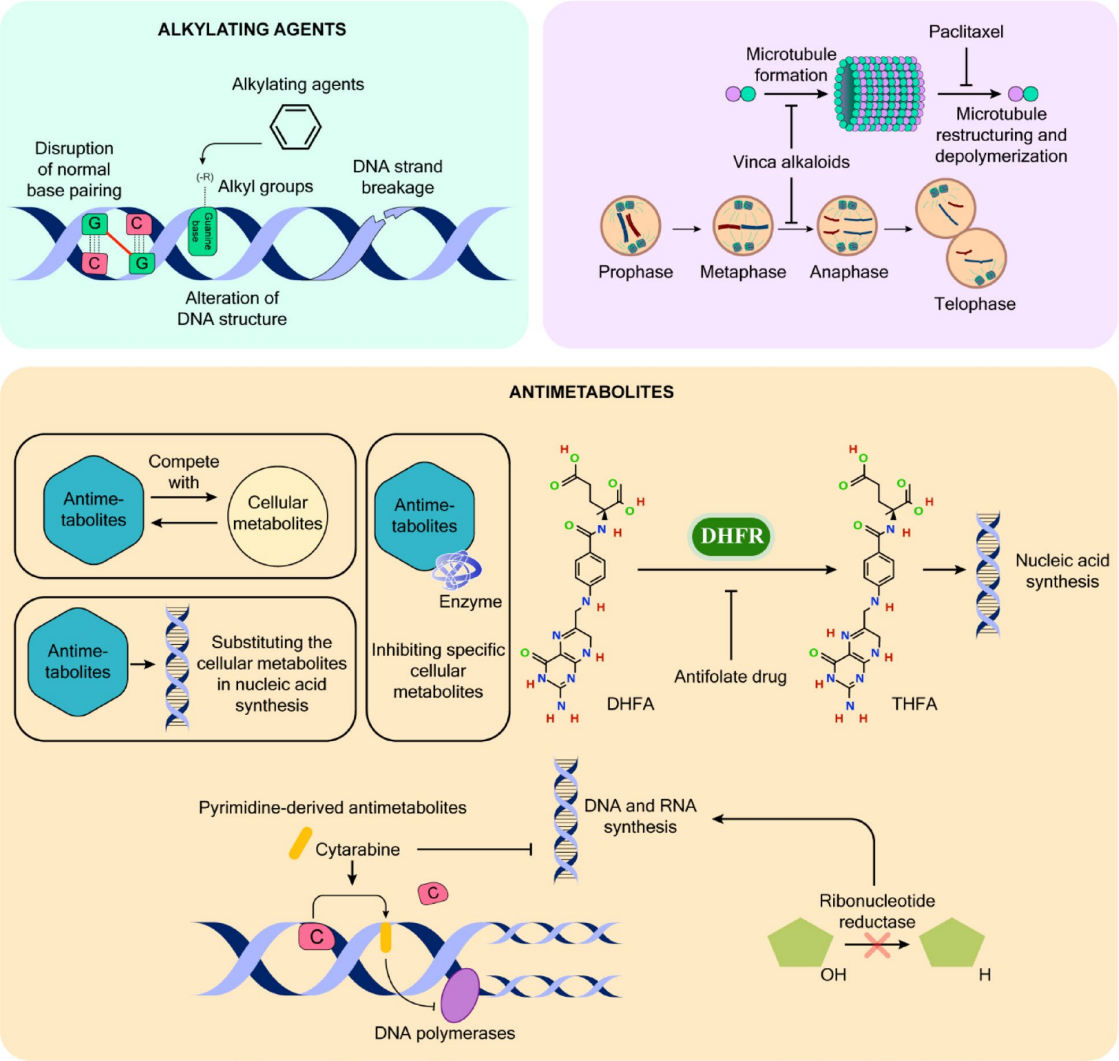
Mechanisms of Action of Common Chemotherapeutic Agents. The figure illustrates the molecular mechanisms by which three major classes of chemotherapeutic drugs exert their anticancer effects: alkylating agents, antimicrotubule agents, and antimetabolites. Alkylating agents modify DNA by adding alkyl groups to guanine bases, leading to base mispairing, DNA strand breaks, and structural disruption, thereby impairing replication and transcription. Antimicrotubule agents, including vinca alkaloids and paclitaxel, disrupt mitotic spindle dynamics. Vinca alkaloids inhibit microtubule polymerization, arresting cells at metaphase, whereas paclitaxel stabilizes microtubules and prevents their depolymerization, ultimately blocking cell division during mitosis. Antimetabolites interfere with nucleotide synthesis and DNA replication. These drugs mimic endogenous metabolites, competing with or replacing them in critical biosynthetic pathways. Antifolate drugs inhibit dihydrofolate reductase (DHFR), blocking the conversion of DHFA to THFA, key cofactors in nucleic acid synthesis. Pyrimidine analogs such as cytarabine are incorporated into DNA, inhibiting DNA polymerases and halting replication. Other agents inhibit enzymes like ribonucleotide reductase, reducing the availability of deoxyribonucleotides for DNA synthesis. Together, these agents target rapidly dividing cells through distinct but complementary mechanisms, highlighting their utility in cancer chemotherapy. The figure was created in Adobe Illustrator

Paclitaxel, a complex drug containing a diterpene taxane ring structure, is extracted from the Western yew tree (*Taxus brevifolia*) and exhibits cytotoxic effects through a unique mechanism [114]. Its cytotoxic activity stems from its ability to prevent microtubule restructuring and depolymerization, processes crucial for tubulin-microtubule dynamics [115]. Clinical studies have demonstrated paclitaxel’s effectiveness in treating metastatic ovarian and breast carcinomas, particularly in cases where initial chemotherapy has failed or relapse has occurred [116]. The drug has also shown promising results in treating advanced-stage tumors, such as breast cancers [86, 117]. Docetaxel, a more potent derivative of paclitaxel, exhibits similar therapeutic actions. It has proven particularly valuable in treating breast cancers that have developed resistance to initial chemotherapy regimens [118]. However, extended exposure to these medications can lead to significant adverse effects, most notably neutropenia, cardiac arrhythmias, neuropathy, and heart failure. Figure 2 illustrates the most common chemotherapeutic agents used in cancer therapy.

### Targeted drug delivery

A significant limitation of conventional chemotherapy is its lack of selectivity in targeting cancer cells, resulting in insufficient drug delivery to tumor sites. Recent decades have witnessed the development and testing of innovative approaches that show promise for future therapeutic strategies. These include nanoparticle-based delivery systems, targeted antibodies, aptamer functionalization, and specific medications like Herceptin for breast cancer treatment [119–121].

Cancer-specific antibodies have emerged as leading candidates for targeted drug delivery, spurring research into more refined and effective approaches [122, 123]. Similarly, various nanocarrier systems and nano-drug formulations have enhanced the precision of drug delivery to cancerous cells [124–126]. The primary advantage of targeted delivery systems, particularly nanomedicine, lies in their ability to minimize drug-induced toxicity in surrounding tissues and organs, thereby reducing collateral damage that often leads to organ stress and failure. However, many of these delivery methods are still undergoing clinical evaluation to determine their effectiveness in patients. More comprehensive information about these delivery systems can be found in previously published literature [127, 128].

### Personalized medication

Cancer exhibits significant patient-to-patient variation, with each case displaying unique characteristics in genetic mutations, development patterns, therapeutic responses, and drug resistance potential. Modern technological advances enable healthcare providers to analyze these individual molecular variations and disease heterogeneity, facilitating the development of personalized treatment strategies [129]. This individualized approach leads to more precise drug selection and dosing protocols, ultimately enhancing treatment effectiveness.

Specific targeted therapies have been developed based on genetic profiles. For example, BRAF mutations can be targeted in melanoma using dabrafenib or vemurafenib [130–132]. This therapeutic approach relies on comprehensive analysis of multiple omics layers (including genomic, transcriptomic, proteomic, and metabolomic data) to identify key molecular drivers of cancer progression and develop targeted interventions.

Research has revealed that certain cancer subtypes frequently display specific mutation patterns. This understanding has led to the development of mutation-specific treatment strategies tailored to individual patients. A notable example is the discovery that approximately 5% of NSCLC patients carry mutations in the anaplastic lymphoma kinase (EML4-ALK) gene, leading to the development of targeted inhibitors like crizotinib and ceritinib [133]. Similarly, vemurafenib was developed as an oral medication specifically designed to target mutant BRAFV600E with higher specificity than wild-type BRAF, making it particularly effective for patients whose tumors harbor this mutation [134]. Clinical basket studies investigated the combination of hormonal therapy with alpelisib (a p110α PIK3CA-specific inhibitor) in breast cancer patients with PIK3CA mutations. These findings highlight the need to screen patients for such molecular changes and implement the proven treatment protocol to enhance tumor response [135]. Research has also linked abnormal FGF pathway signaling to cancer development and progression. Various genetic modifications, including FGF receptor amplification, mutations, and gene fusions, show different responses to treatments, necessitating molecular analysis for optimal drug selection [136, 137]. Another notable advancement is the use of olaparib, a PARP inhibitor, which effectively targets BRCA-mutated cancer cells while minimizing impact on healthy tissue [138, 139].

In terms of cell protection, scientists have discovered that 2,3-Dihydro-3beta-methoxy withaferin-A, a naturally occurring alkylated withanolide, helps shield normal cells from various stresses associated with cancer treatments [140]. While precision medicine has proven beneficial for many patients, the high costs of genomic profiling technologies make these modern therapeutic approaches inaccessible to much of the global population [141].

### Synergistic and combination therapies

**Fig. 3:**
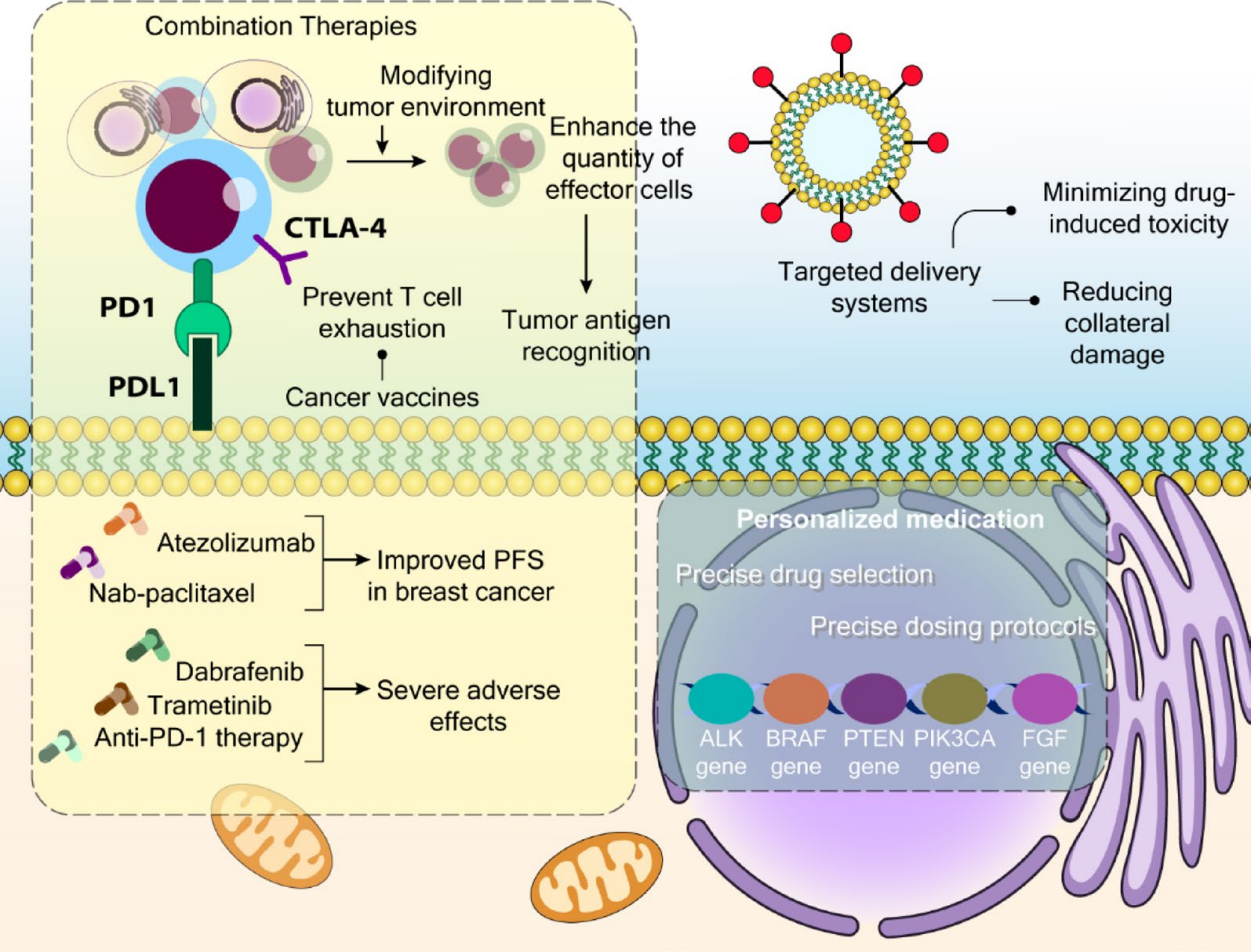
Targeted Drug Delivery and Combination Therapy to Improve Cancer Chemotherapy. Advanced approaches in cancer treatment, focusing on immunotherapy, targeted delivery, and personalized medicine, are illustrated here. Combination therapies enhance immune system engagement by preventing T cell exhaustion through checkpoint inhibitors such as anti-PD-1/PD-L1 and anti-CTLA-4 antibodies. These strategies aim to strengthen tumor antigen recognition, increase the number and activity of effector T cells, and modify the tumor microenvironment to support immune infiltration. Cancer vaccines also contribute by priming the immune system against tumor-associated antigens. Clinical examples include atezolizumab combined with nab-paclitaxel, which has demonstrated improved progression-free survival (PFS) in breast cancer, and combination regimens involving dabrafenib, trametinib, and anti-PD-1 therapy, which, although effective, have been associated with significant toxicity. Targeted drug delivery systems, such as nanoparticles, offer site-specific transport of therapeutic agents, aiming to minimize systemic toxicity and reduce damage to healthy tissues. Personalized medicine incorporates genomic profiling to guide therapy decisions. By identifying key genetic mutations (e.g., in ALK, BRAF, PTEN, PIK3CA, and FGF genes), clinicians can tailor drug selection and dosing protocols, enhancing therapeutic precision while minimizing adverse effects. The figure was created in Adobe Illustrator

The integration of targeted therapies with immunotherapy presents a promising approach, potentially combining rapid tumor reduction with sustained immune responses (Fig. 3) [142]. This dual strategy could help establish long-term remission by enabling the immune system to target multiple antigens, thereby reducing the likelihood of drug-resistant cancer cells emerging [143]. One effective strategy involves pairing cancer vaccines with ICIs that target cytotoxic T-lymphocyte–associated protein 4 (CTLA-4) and programmed cell death protein 1 (PD-1)/programmed death-ligand 1 (PD-L1), which helps prevent T cell exhaustion [144, 145]. Additionally, certain targeted medications can modify tumor blood vessels, enabling better infiltration of immune cells, including T-cells and NK-cells [146].

Consider gemcitabine, a deoxycytidine analog that promotes tumor cell death, leading to enhanced antigen presentation and immune activation [147]. In metastatic NSCLC treatment, physicians now routinely prescribe pembrolizumab alone, or in combination with carboplatin and pemetrexed, as first-line therapy [148]. For patients with triple-negative breast cancer, a new treatment combining nab-paclitaxel and atezolizumab has been approved, showing improved PFS compared to standard chemotherapy [149].

Adenosine receptors (ARs) play a crucial role in cancer progression [150]. Scientists have identified four ARs (A_1_, A_2A_, A_2B_, and A_3_), all belonging to the GPCR class A family [151]. While A2BR shows promise for chemotherapy applications [152], A2AR is particularly relevant for immunotherapy approaches [153, 154]. Some compounds that target both A2AR and A2BR have emerged as potential candidates for combined chemo-immunotherapy strategies [155, 156].

Epigenetic regulators serve a dual purpose in the tumor environment by enhancing both antigen expression and T cell function [157]. The restoration of exhausted T cells through ICI treatment involves significant chromatin changes, suggesting that epigenetic therapy might benefit patients with impaired T cell function [158].

One innovative therapeutic approach diverges from modifying the existing tumor environment, instead focusing on introducing engineered immune cells or cellular receptors through adoptive cellular therapy. This method aims to enhance the quantity of effector cells capable of tumor antigen recognition [159]. Laboratory-cultured lymphocytes may exhibit stronger anticancer responses upon reintroduction to the patient, as they haven’t been exposed to the suppressive signals within the tumor microenvironment [160]. Currently, researchers are exploring three primary types of adoptive T cell therapy: TIL therapy [161], CAR T-cell therapy [162, 163], and TCR-engineered cell therapy [164, 165].

Another strategy involves the direct administration of oncolytic viruses into tumors to enhance antigen recognition and T cell activity [166]. For instance, T-VEC, a modified herpes virus, is administered directly into melanoma lesions when surgical removal isn’t possible. This treatment triggers immediate tumor cell death and stimulates GM-CSF production, which attracts and activates APCs [167].

Recent drug development has adopted a more holistic approach to the TME, exploring methods to simultaneously boost immune function and create therapeutic synergy [168, 169]. Moving forward, research should continue investigating the complex relationships between targeted and immune therapies, while optimizing treatment parameters such as timing, dosing, and sequence to maximize therapeutic benefits.

## The natural bioactive-chemotherapeutics synergism and related signaling mechanisms

When multiple components interact to produce effects that differ from their individual impacts, this is known as pharmacological synergy or synergism, particularly in the context of whole-plant effects rather than isolated active ingredients. This phenomenon shares similarities with potentiation, where two simultaneously administered substances produce combined effects that exceed the sum of their individual actions [170]. The primary advantage of synergistic approaches lies in their ability to achieve therapeutic outcomes with lower combined doses compared to higher individual doses. Ideally, this reduced dosing strategy should maintain effectiveness comparable to single-drug treatments while minimizing adverse effects [171]. The principle of synergy is fundamental to combination therapy, which has shown significant success in treating complex conditions including cancer, hypertension, and asthma, leading to improved patient outcomes [172]. For example, in cancer treatment, combining multiple drugs that target either cancer cells or their associated pathways has strengthened therapeutic strategies. This multi-drug approach yields positive clinical results by preventing drug resistance through the simultaneous application of synergistic toxic effects from multiple compounds [173].

The past two decades have witnessed increasing integration of traditional medical treatments with complementary therapeutic approaches, encompassing various disciplines including phytotherapy, gemmotherapy, pharmacognosy, ethnopharmacology, and herbal medicine. When combining natural substances with standard chemotherapy drugs, practitioners aim to enhance treatment effectiveness while decreasing harmful side effects [170]. Clinical and pharmacological research has identified four primary synergistic mechanisms. Individual compounds or plant-based mixtures can affect various cellular targets beyond their expected scope. Changes in fundamental properties such as solubility may occur. Natural components can help counteract the development of drug resistance. Certain compounds or plant extracts can diminish or counteract a drug’s harmful effects, thereby minimizing adverse reactions [170]. While combination therapy is no longer novel in modern medicine, the discovery of new additive interactions that enhance current medical treatments continues to spark interest.

### Natural compounds synergistically enhance the effects of cancer chemotherapy

**Fig. 4:**
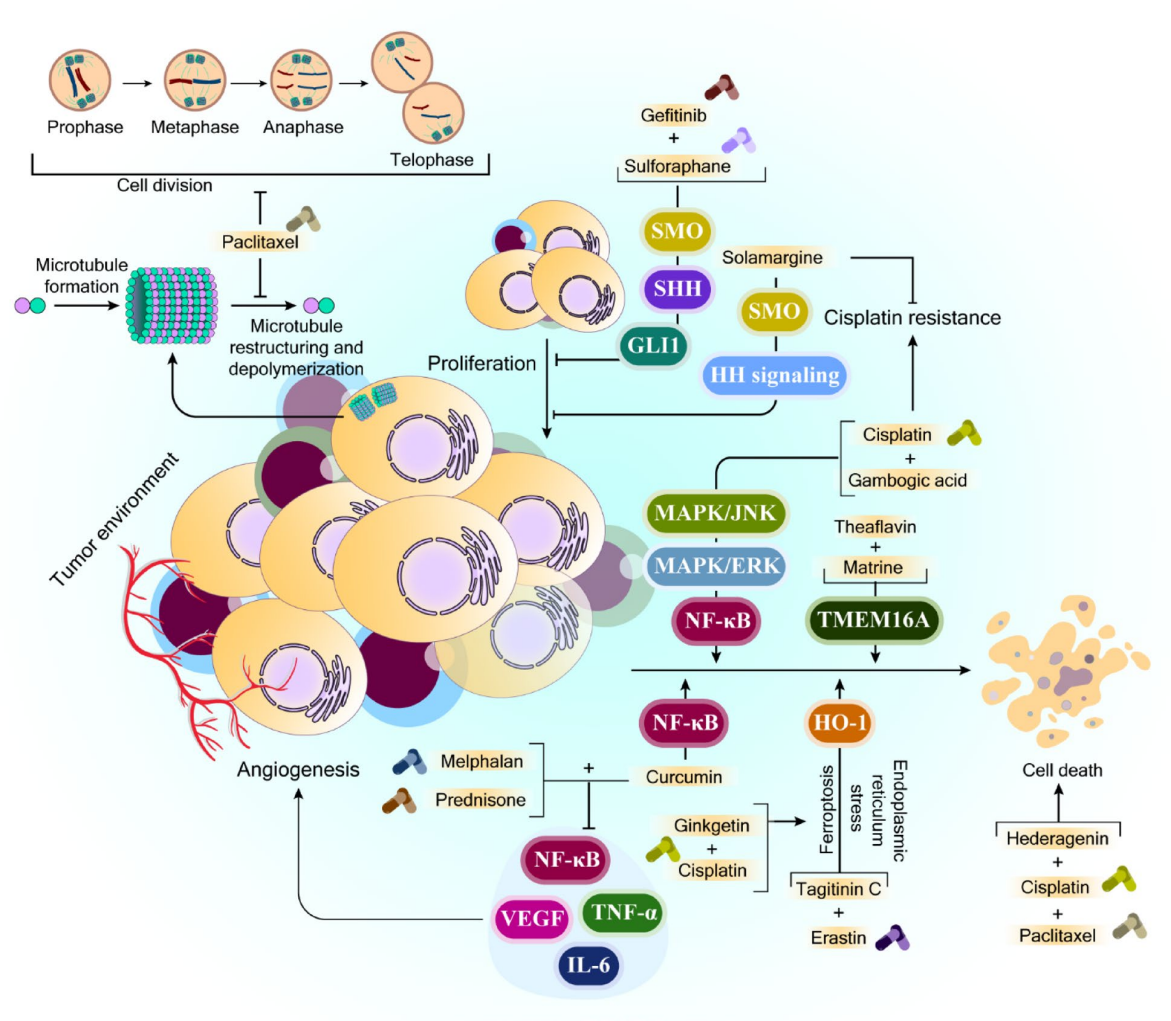
Signaling Mechanisms by which Natural Compounds Synergistically Enhance the Effects of Cancer Chemotherapy. This schematic illustrates the complex interplay between signaling pathways and pharmacological interventions regulating cancer cell proliferation, angiogenesis, chemoresistance, and cell death. The process of cell division is shown, with microtubule dynamics being a critical target of the chemotherapeutic agent paclitaxel, which disrupts microtubule restructuring and depolymerization, thereby inhibiting mitotic progression and halting proliferation. Tumor growth is driven by proliferative signals within the tumor microenvironment, including aberrant activation of Hedgehog (HH) signaling via SHH, SMO, and GLI1, contributing to chemoresistance. Agents such as gefitinib, sulforaphane, and solamargine target components of this pathway to reduce tumor cell survival and overcome cisplatin resistance. Additional resistance mechanisms involve activation of MAPK/ERK, MAPK/JNK, and NF-κB signaling, which can be suppressed by natural compounds such as gambogic acid, theaflavin, and matrine. TMEM16A contributes to resistance and survival by modulating stress pathways, while inhibition of HO-1 can trigger ferroptosis and endoplasmic reticulum stress, promoting cancer cell death. Combinatorial treatments (e.g., tagitinin C and erastin) enhance these cytotoxic effects. Tumor angiogenesis is mediated by pro-inflammatory cytokines and growth factors, including NF-κB, VEGF, TNF-α, and IL-6. Drugs such as melphalan and prednisone suppress angiogenesis and inflammation, while curcumin and ginkgetin—used with cisplatin—target inflammatory mediators to inhibit tumor progression. Finally, combination chemotherapies (e.g., cisplatin with paclitaxel or hederagenin) enhance cell death, demonstrating how multi-targeted regimens can overcome resistance and induce apoptosis. The figure was created in Adobe Illustrator

Recent advances in cancer therapeutic research have led to the emergence of combination therapy approaches, utilizing chemotherapeutic drugs that target different pathways to combat tumor progression. Due to the considerable side effects associated with chemotherapy, researchers are exploring new treatment strategies that combine traditional chemotherapeutic agents with less harmful natural compounds (Fig. 4).

The transcription factor NF-κB, originally identified in B cells, recognizes the κ light chain enhancer sequence and is crucial for cellular functions including proliferation, survival, and migration [174]. Research has established NF-κB activation as a significant cancer indicator, particularly in prostate and bladder malignancies [175, 176]. The interaction between chronic inflammation and NF-κB can enhance tumor development through multiple mechanisms, including reactive oxygen species-induced DNA damage and inflammatory mediator stimulation. This suggests that NF-κB could be targeted by natural compounds to enhance chemotherapy effectiveness [177].

The natural yellow pigment curcumin, derived from *Curcuma longa* (turmeric) roots, has demonstrated various biological activities, particularly in cancer cell growth inhibition and apoptosis induction [178]. Studies have revealed curcumin’s ability to trigger cancer cell death through NF-κB suppression in various cell types, including MCF-7 breast cancer cells, pancreatic stellate cells, and hepatic cancer stem cells [179–181]. Research indicates curcumin may enhance the effectiveness of paclitaxel in ovarian cancer by regulating the miR-9-5p/BRCA1 axis [182]. A clinical trial demonstrated that combining curcumin with melphalan and prednisone in transplant-ineligible multiple myeloma patients effectively reduced NF-κB activation, vascular endothelial growth factor (VEGF), TNF-α, and IL-6 levels, resulting in improved patient outcomes [183]. Garcinia hanburyi tree resin yields gambogic acid (GA) [184], which works synergistically with cisplatin in NSCLC treatment. This combination enhances cisplatin’s effectiveness by blocking NF-κB subunits (p65 and p50), mitogen-activated protein kinase (MAPK)/ERK, and MAPK/JNK pathways, promoting apoptosis in A549 and NCI-H460 cancer cells [185].

The protein Hedgehog (HH), first identified in *Drosophila melanogaster*, plays an essential role in cellular processes including growth, differentiation, and survival [186]. Two main theories explain its involvement in cancer development upon abnormal activation. The first theory proposes that HH signaling directly affects tumor cell survival and proliferation, particularly through its influence on Warburg-like glycolytic metabolism [187]. The second theory suggests that HH signaling impacts tumor development indirectly by stimulating surrounding stromal cells through paracrine signaling [188]. The traditional Chinese medicinal plant *Solanum nigrum* L. contains solamargine, a steroidal alkaloid with documented anti-inflammatory and anticancer properties [189]. Studies have shown that solamargine counteracts cisplatin resistance in NSCLC by targeting SMO and inhibiting HH signaling, leading to reduced cell proliferation and increased cell death. Notably, combining solamargine with cisplatin produced enhanced therapeutic effects [190]. Cruciferous vegetables contain sulforaphane, an isothiocyanate that induces G2/M phase cell cycle arrest and cell death in colon cancer cells [191]. When combined with gefitinib, sulforaphane shows dose-dependent inhibition of SHH, SMO, and GLI1 expression, effectively suppressing the growth of gefitinib-resistant lung cancer cells through SHH pathway modulation [192].

Chemotherapeutic drugs often act by inducing mitochondrial reactive oxygen species (ROS) production to cause cellular damage. However, cancer cells can activate autophagy to reduce ROS levels, diminishing the effectiveness of chemotherapy, such as in breast cancer [193]. This has led researchers to explore strategies targeting autophagic flux inhibition, which results in damaged mitochondria accumulation and increased ROS levels, ultimately promoting cancer cell death [194]. Hederagenin, a pentacyclic triterpenoid present in various medicinal plants, exhibits multiple therapeutic properties including anticancer, anti-inflammatory, and antidepressant effects [195]. Research by Wang et al. demonstrated that hederagenin inhibits autophagy in lung cancer cells by enhancing LC3-I to LC3-II conversion. When combined with either paclitaxel or cisplatin, hederagenin enhanced their anticancer efficacy, demonstrating synergistic effects [196].

The Nrf2/HO-1 signaling pathway plays a crucial role in maintaining cellular redox balance and homeostasis in mammals. Research has demonstrated that this pathway, which is linked to ferroptosis, significantly influences tumor cell apoptosis. Among the bioactive compounds from *Tithonia diversifolia*, tagitinin C exhibits diverse therapeutic properties, including anti-inflammatory and anti-glioblastoma effects. When combined with erastin, it enhances apoptosis in HCT116 cells through increased endoplasmic reticulum stress and ferroptosis activation. While erastin induces ferroptosis by blocking the cystine-glutamate antiporter, tagitinin C facilitates cell death through HO-1 upregulation, promoting iron and ROS accumulation. However, conflicting findings exist regarding this pathway’s therapeutic effectiveness. Some research indicates that using inhibitors of Nrf2-associated proteins during chemotherapy can enhance treatment outcomes [197–200], contradicting Tagitinin C’s observed effects. For instance, Ginkgetin, extracted from Ginkgo biloba, works synergistically with cisplatin by promoting ferroptosis, enhancing ROS generation, and suppressing Nrf2/HO-1 in NSCLC [201]. Given the pathway’s complex role in oxidative stress and cellular detoxification, coupled with ongoing debates about HO-1’s mechanism, further investigation is required to fully eluicidate Nrf2/HO-1 as a therapeutic target.

TMEM16A, a calcium-activated chloride channel, maintains ionic homeostasis and shows elevated expression in various malignancies including prostate, lung, and colorectal cancers. Studies have revealed that TMEM16A suppression reduces tumor progression, enhances chemosensitivity, and extends patient survival [202]. The citrus-derived flavonoid narirutin potentiates cisplatin’s anticancer effects through TMEM16A inhibition [203]. Similarly, Homoharringtonine, derived from Cephalotaxaceae, demonstrates clinical anticancer efficacy [204] and inhibits TMEM16A dose-dependently, showing significant anti-lung cancer properties in ex vivo studies [205]. Additionally, both theaflavin from black tea and matrine have shown anti-lung cancer potential through TMEM16A modulation [206, 207]. As a recently identified cancer target, TMEM16A offers promising therapeutic potential due to its safety profile and limited toxicity. The combination of TMEM16A inhibitors with conventional chemotherapy could represent an innovative approach for treating cancers with high TMEM16A expression.

### **The mechanisms by which natural compounds mitigate tumor drug resistance**

Drug resistance in cancer treatment represents a critical challenge, with research indicating it accounts for over 90% of cancer-related deaths through various complex mechanisms [208]. Several key mechanisms contribute to chemotherapy resistance: First, cancer cells utilize P-glycoprotein to pump chemotherapeutic agents out of cells, thereby reducing their internal concentration and effectiveness [209]. Second, these cells enhance their DNA repair capabilities through processes such as nucleotide and base excision repair and mismatch repair pathways, helping them survive platinum-based treatments [210, 211]. Third, alterations in crucial genes, including EGFR and other drug targets, can render chemotherapeutic agents ineffective [212, 213].

Platelet-activating factor receptor (PAFR), as a G protein-coupled receptor, has recently been identified with roles extending beyond platelet aggregation to inflammatory and immune responses in a group of malignancies, particularly skin cancer [214]. Recent studies have demonstrated PAFR’s significant involvement in cancer progression, with elevated expression noted in various malignancies including prostate and ovarian cancers [215, 216]. In non-small cell lung cancer, PAFR establishes a self-reinforcing cycle with STAT3, promoting tumor progression and metastasis [217]. Studies of esophageal squamous carcinoma have revealed that PAFR promotes cancer advancement through PI3K/AKT pathway activation [218]. Additionally, research by Aponte and colleagues demonstrated that PAF/PAFR signaling enhances ovarian cancer growth and invasion via the tyrosine phospho-EGFR/Src/FAK/paxillin pathway [215].

Ginkgolide B, extracted from the *Ginkgo biloba*, is nature’s most potent PAFR antagonist [219]. Studies show that combining ginkgolide B with gemcitabine at non-toxic concentrations enhances gemcitabine’s effectiveness against resistant pancreatic cancer cells. This improvement occurs through the suppression of the PAFR/NF-κB pathway, ultimately reducing gemcitabine resistance in pancreatic cancer [220]. PAFR’s crucial role has also been validated in oral cancer; combining cisplatin with ginkgolide B has shown reduced PAFR activity and decreased phosphorylation in the ERK and Akt pathways. This combination therapy enhanced cell death through increased cleaved caspase-3 expression, making oral cancer cells more responsive to cisplatin [221]. Similar therapeutic benefits were observed in ovarian cancer, where this combination approach effectively decreased tumor growth and enhanced drug efficacy [222]. Ichim and colleagues highlighted the dual nature of chemotherapy and radiotherapy-induced apoptosis, noting its potential to both eliminate cancer cells and paradoxically stimulate tumor development [223]. Recent discoveries have revealed that cancer treatments involving chemotherapy and radiotherapy generate PAF, which exhibits cancer-promoting properties when it interacts with PAFR. These findings suggest that natural PAFR inhibitors could represent an innovative cancer treatment approach [224].

Prolyl isomerase 1 (Pin1), which catalyzes peptidyl-prolyl cis-trans isomerization, influences protein function through structural modifications and plays significant roles in a variety of diseases, including Alzheimer’s and various cancers [225]. Beyond activating multiple cancer-related pathways (including Raf/MEK/ERK, PI3K/Akt, Wnt/β-catenin, and NF-κB), Pin1 also contributes to drug resistance, such as in breast cancer, through inducing the LC-3 expression and mediating tamoxifen resistance [226]. Research by Koikawa et al. demonstrated elevated Pin1 expression in pancreatic ductal adenocarcinoma and associated fibroblasts. Their study showed that combining Pin1 inhibitors with PD1 inhibitor αPD1 enhanced cell death and substantially reduced tumor growth in both human and KPC PDAC-like organoids in GDA mice [227]. These findings highlight Pin1 inhibition as a promising therapeutic strategy for this aggressive cancer type.

Juglone, a natural compound from walnut trees (*Juglans* spp.) and its derivatives function as Pin1 inhibitors, showing promise for reducing cancer treatment resistance [228]. Research by Sajadimajd et al. demonstrated that in trastuzumab SKBR3 cells, juglone triggered cell death, suppressed cellular growth, colony development, and movement, while combating drug resistance through Pin1 and Notch1 inhibition [229]. Complementary findings by Yun et al. revealed juglone enhances trastuzumab’s effectiveness in metastatic breast cancer BT474 cells by increasing FAS reduction and apoptosis. The combination of trastuzumab with either juglone or gene silencing led to increased cleaved poly(ADP-ribose) polymerase and DNA fragmentation, improving trastuzumab sensitivity [230]. For estrogen receptor alpha-positive breast cancer, juglone shows dose-dependent inhibition of TPA-induced tumor cell transformation by counteracting TPA-induced E2F-4 and Egr-1 increases and reducing LC-3, thus making tamoxifen-resistant MCF-7 cells more responsive to treatment [226]. Other effective Pin1 inhibitors that help reduce tumor resistance include EGCG, all-trans retinoic acid (ARTA), and arsenic trioxide (ATO) [231].

Similar resistance mechanisms are exhibited by multidrug resistance (MDR)-associated proteins 1 and 2, and breast cancer resistance protein, which are considered primary contributors to MDR through enhanced chemotherapeutic drug efflux [232]. Schisandrin B, derived from the traditional Chinese medicine *Schisandra chinensis*, exhibits antioxidant and antitumor properties [233]. This compound reduces tumor drug resistance by decreasing p-glycoprotein expression across various cancer types [234]. In doxorubicin-resistant breast and ovarian cancer cells, schisandrin B suppresses P-glycoprotein expression and activity, increasing intracellular doxorubicin accumulation and reducing resistance [235]. Studies have shown that Schisandrin B can reverse resistance to chemodrugs in cancer cells through direct inhibition with p-glycoprotein [236]. Teng et al. found that caffeic acid, a common plant phenolic acid, reverses tumor cell resistance to vincristine, paclitaxel, and doxorubicin while increasing apoptosis [237]. Recent research shows that glabratephrin, a prenylated flavonoid from *Tephrosia purpurea*, enhances doxorubicin effectiveness in triple-negative breast cancer cells by reducing doxorubicin affinity for P-glycoprotein and preventing efflux, without affecting P-glycoprotein expression [238]. This p-glycoprotein resistance reversal mechanism represents a promising direction for developing chemotherapeutic sensitizers and offers a potentially safe and effective approach for treating drug-resistant tumors.

PI3K/Akt pathway serves as a crucial cellular signaling mechanism, particularly in regulating glucose uptake and metabolism [239]. In cancers like breast and ovarian malignancies, this pathway’s hyperactivation and mutations in PIK3CA gene creates optimal conditions for tumor growth and proliferation, contributing significantly to drug resistance development [240]. Quercetin, a polyphenolic flavonoid abundant in fruits and vegetables, demonstrates multiple therapeutic properties including anti-inflammatory and antioxidant effects [241]. Studies using both in vivo and in vitro models of docetaxel resistance have shown that combining quercetin with docetaxel enhances the suppression of cell proliferation, metastasis, and invasion, while countering docetaxel resistance through PI3K/AKT pathway modulation in prostate cancer [242]. Similar therapeutic benefits have been observed with isorhamnetin, another flavonoid compound related to quercetin, in prostate cancer [243]. Toosendanin, a triterpenoid from *Melia toosendan* Sieb. et Zucc. with known anthelmintic and antimicrobial properties [244], shows promise in cancer therapy. At non-toxic levels, toosendanin significantly enhanced apoptosis in Adriamycin-resistant MCF-7 cells and inhibited PI3K [245]. While individual treatments showed limited effectiveness, the combination therapy achieved a remarkable 90% reduction in tumor volume. Matrine, the primary active component from *Sophora flavescens* (matrine plant), reduces MCF-7 cell drug resistance through dual mechanism: PI3K/AKT upregulation and AKT phosphorylation reduction via PTEN [246]. Apigenin, a flavonoid with diverse biological activities (incl. antioxidant, anti-inflammatory, anti-hepatic lipid accumulation, anticancer, and neuroprotective effects) [247], shows effectiveness against gemcitabine-resistant pancreatic cancer. When used in combination with gemcitabine, apigenin disrupts the cell cycle in resistant cells, reduces gemcitabine-induced p-Akt levels, and triggers tumor cell apoptosis [248].

Recent studies identify epithelial-mesenchymal transition (EMT) as critical for cancer cell drug resistance development. This resistance develops primarily through increased drug transporter expression (including P-glycoprotein and multidrug resistance-associated protein 1) and suppression of apoptotic pathways [249]. The Notch signaling pathway plays a vital role in EMT and drug resistance development. Studies of breast cancer cells with elevated Notch expression show that Notch IC activation upregulates SLUG, leading to E-cadherin suppression and subsequent EMT progression [250]. This pathway’s involvement in drug resistance has been documented across various cancers, including prostate and lung cancers [251, 252]. Natural compounds like curcumin, which act as Notch inhibitors, have shown promise as complementary treatments targeting cancer cells and stem cells in cancers such as cervical and oral carcinomas [253, 254]. The TGF-β pathway represents another crucial EMT mechanism, significantly influencing cancer heterogeneity and drug resistance in squamous cell carcinomas [255]. Research has shown that MHP-1, a novel polysaccharide extracted from Mortierella hepialid, reduces topiramate resistance in breast cancer cells by suppressing EMT through TGF-β pathway inhibition [85].

While many chemotherapeutic agents kill cancer cells by inducing DNA damage, these cells often develop resistance through enhanced DNA repair mechanisms. O(6)-methylguanine-DNA methyltransferase (MGMT) serves as a key transferase in DNA repair, removing toxic and premutagenic O6-methylguanine DNA adducts [256]. Lipoic acid, a natural disulfide-containing mitochondrial enzyme cofactor, has been shown to enhance the effectiveness of the alkylating agent N-methyl-N-nitrosourea by inhibiting MGMT and reducing temozolomide resistance in HCT116 colorectal cancer cells [257].

**Fig. 5:**
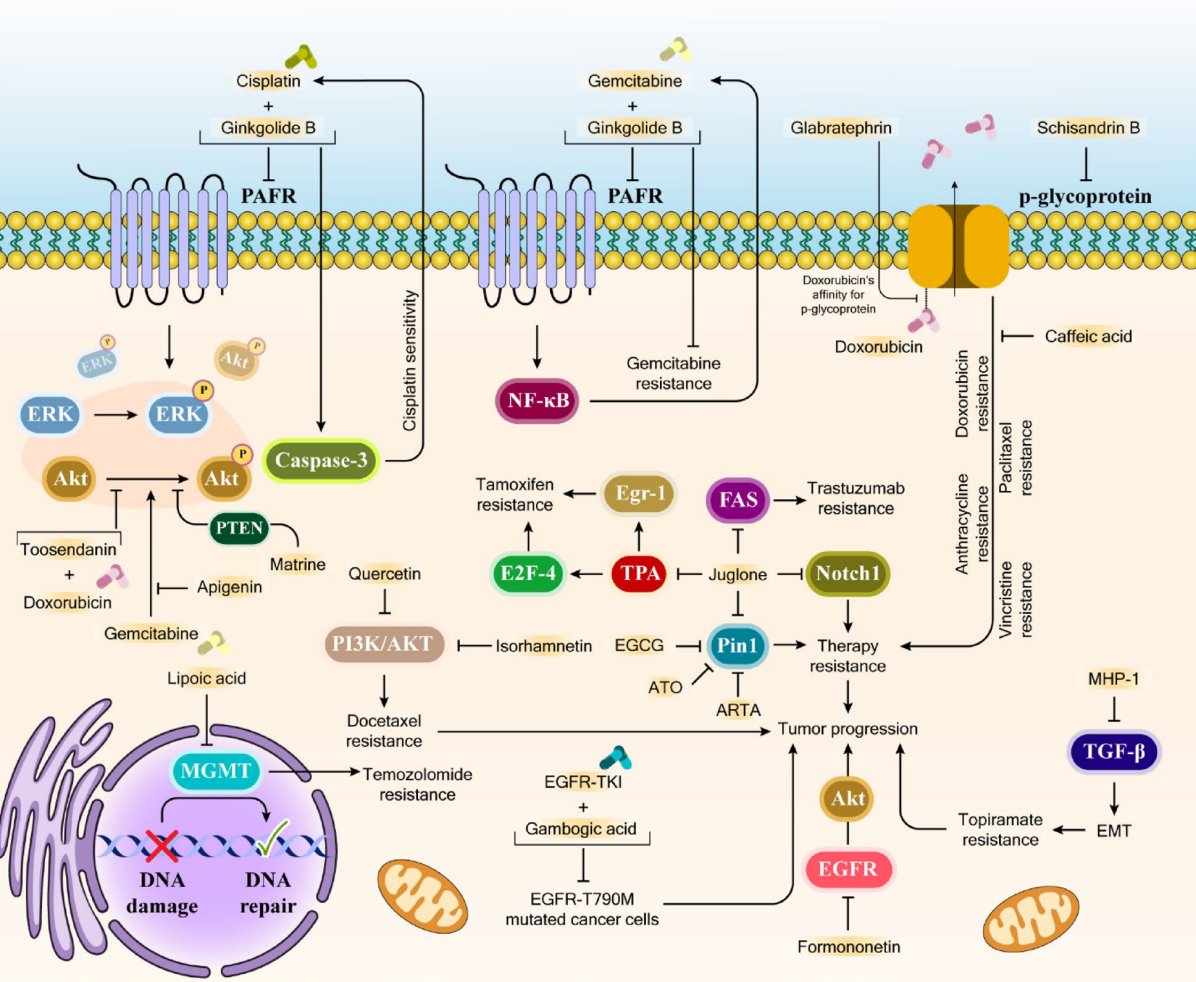
Signaling Mechanisms by Which Natural Compounds Mitigate Tumor Drug Resistance. This schematic illustrates molecular pathways involved in cancer drug resistance and how natural compounds modulate them to restore therapeutic sensitivity. Key mechanisms depicted include: Efflux Pump Regulation: Shows P-glycoprotein’s role in resistance to doxorubicin, paclitaxel, vincristine, and anthracyclines, and how glabratephrin, schisandrin B, and caffeic acid influence drug affinity or inhibit efflux. Signaling Pathway Modulation: Several pathways such as ERK, Akt, PI3K/AKT, NF-κB, and EGFR are shown to be involved in drug resistance (e.g., gemcitabine, docetaxel, tamoxifen, trastuzumab). Natural compounds like ginkgolide B, apigenin, matrine, quercetin, isorhamnetin, EGCG, formononetin, and gambogic acid are depicted as modulators of these pathways, affecting sensitivity to various chemotherapeutics or targeting specific resistant cell types (e.g., EGFR-T790M mutated cancer cells). DNA Damage Response and Repair: The involvement of MGMT in temozolomide resistance through DNA repair mechanisms is illustrated, along with the influence of lipoic acid. Apoptosis and Cell Cycle Regulation: The role of Caspase-3, PTEN, Egr-1, FAS, and Notch1 in therapy resistance and tumor progression is highlighted, with compounds like juglone, TPA, and Pin1 modulators impacting these processes. EMT: The pathway involving MHP-1 and TGF-β leading to EMT and topiramate resistance is also included. The figure was created in Adobe Illustrator

The epidermal growth factor receptor (EGFR) promotes cancer cell survival, proliferation, and invasion through wild-type signaling. While EGFR-tyrosine kinase inhibitors (TKI) like gefitinib target EGFR, mutations such as T790M or S492R often lead to treatment resistance [258]. Studies have demonstrated that combining gambogic acid with EGFR-TKI effectively suppresses EGRF-T790M mutated lung adenocarcinoma cells both in vitro and in vivo [259]. Furthermore, formononetin, another EGFR inhibitor, has shown effectiveness in non-small cell lung cancer treatment by binding to both wild-type and mutant EGFR ATP-binding pockets, inhibiting EGFR-Akt signaling, and promoting Mcl-1 degradation through ubiquitination [260]. In Fig. 5, you can see key mechanisms through which natural bioactives decrease cancer drug resistance. Furthermore, Table 2 lists the most substantial natural compounds enhancing chemotherapy efficacy and overcoming drug resistance in cancer.

**Table 2:** Natural compounds enhancing chemotherapy efficacy and overcoming drug resistance in cancer

Natural compound	Chemotherapeutic agent	Mechanism of action	Cancer type	References
Curcumin	-	Inhibits NF-κB, enhances apoptosis, reduces inflammation	Breast cancer	[179]
Gambogic Acid	Cisplatin	Inhibits NF-κB and MAPK/HO-1 signalling	NSCLC	[185]
Solamargine	Cisplatin	Not defined (phenotypic screening)	NSCLC	[190]
Sulforaphane	Gefitinib	Regulation of SHH signaling	Lung cancer	[192]
Hederagenin	Paclitaxel and Cisplatin	Impairing autophagy (LC3-I to LC3-II conversion)	Lung cancer	[196]
Ginkgetin	Cisplatin	Promotes ferroptosis-mediated disruption of Nrf2/HO-1	EGFR wild-type NSCLC	[201]
Narirutin	Cisplatin	Regulation of TMEM16A	Lung cancer	[203]
Ginkgolide B	Cisplatin Gemcitabine	PAFR pathway PAFR/NF-κB pathway	Oral cancer Pancreatic cancer	[220, 221]
Juglone	Trastuzumab	Down-regulation of Notch1 signaling pathway	SKBR3 breast cancer cells	[229]
Schisandrin B	Doxorubicin	Inhibiting P-glycoprotein and promoting proteasome-mediated degradation of survivin	Various cancers	[235]
Caffeic Acid	Vincristine, Paclitaxel	Inhibiting Efflux Function of P-glycoprotein	Various cancers	[237]
Quercetin	Docetaxel	Regulation of androgen receptor and PI3K/Akt signaling pathways	Prostate cancer	[242]
Toosendanin	Adriamycin	Inhibiting PI3K	Breast cancer	[245]
Gambogic Acid	EGFR-TKI (Gefitinib)	Targeting the EGFR-T790M mutation	Lung cancer	[259]

### Natural compounds and side effects of chemotherapy

The effectiveness of cancer treatment is significantly compromised by severe adverse reactions to chemotherapy, with some patient fatalities directly linked to these side effects. Various natural compounds, when used alongside chemotherapeutic drugs, have demonstrated potential in minimizing these adverse effects while maintaining therapeutic efficacy.

Oxaliplatin functions as an anticancer agent by creating DNA-platinum adducts that prevent cancer cell multiplication; among chemotherapy drugs, paclitaxel and oxaliplatin are notorious for causing the most severe peripheral neuropathy [261, 262]. Research indicates that mitochondrial dysfunction plays a central role in developing this neurological complication [263]. Studies have revealed that tanshinone IIA, extracted from the traditional Chinese medicinal herb Salvia miltiorrhiza, protects mitochondria by preventing oxaliplatin-induced ROS elevation in N2a mouse neuroma cells. Tanshinone IIA reduces oxaliplatin-induced peripheral neuropathy by stimulating autophagy through the PI3K/Akt/mTOR pathway. At non-toxic concentrations, tanshinone IIA diminishes oxaliplatin’s pro-apoptotic effects on N2a cells and lessens neurotoxicity in rat models [264].

The natural antioxidants thymoquinone and geraniol show promise in reducing cisplatin-induced neurotoxicity. These compounds work by suppressing apoptosis-related proteins (including p53 and MAPK) while maintaining cisplatin’s anticancer effectiveness against MCF-7 breast cancer cells [265]. Additionally, the isoquinoline alkaloid berberine has demonstrated protective effects against doxorubicin-induced neuroinflammation by enhancing brain AchE activity and reducing oxidative stress-induced neuronal death [266]. Research has also identified that Red ginseng-derived ginsenoside Rg3, a compound classified as a tetracyclic triterpene saponin, has specific inhibitory effects on cancer cell invasion and spread [267].

Gastrointestinal disturbances, particularly diarrhea, represent a common adverse effect of various chemotherapy medications including 5-FU, irinotecan, and celecoxib [268–270]. While mild chemotherapy-related diarrhea may disrupt treatment, severe cases can lead to dehydration, electrolyte imbalance, and nutritional deficiencies, contributing to early mortality in ~ 5% of cancer patients.

The naturally occurring flavonoid hesperidin, abundant in various fruits and flowers, exhibits multiple therapeutic properties including anti-inflammatory, antioxidant, cardiovascular protective, and antitumor effects [271]. Studies have demonstrated that hesperidin administration at doses of 20 mg/kg and 100 mg/kg effectively reduced irinotecan-induced diarrhea in CT-26 tumor-bearing immune mice and lowered severe diarrhea risk. Furthermore, hesperidin demonstrated anti-inflammatory effects in intestinal tissue and, notably, when combined with irinotecan, achieved enhanced antitumor efficacy through STAT3 pathway suppression [272].

Anthracyclines, crucial for treating hematologic malignancies and solid tumors, often cause cardiotoxicity that limits treatment [273]. Among natural compounds, calycosin, derived from *Astragalus membranaceus*, exhibits multiple beneficial properties including anti-inflammatory, antioxidant, anticancer, and cardioprotective effects [274]. Calycosin’s cardioprotective mechanism involves suppressing NLRP3-cystatin-1-GSDMD pathway-mediated pyroptosis [275]. Research by Zhai et al. demonstrated calycosin’s ability to reduce doxorubicin-induced cell death and oxidative stress in H9c2 cells via the Sirtuin-NLRP3 pathway [276]. In zebrafish studies, calycosin demonstrated protective effects against doxorubicin-induced cardiotoxicity via autophagy regulation [277].

The lily family *Colchicum* produces colchicine, an alkaloid also found in various plant parts including corms, seeds, and flowers [278]. Another chemotherapy drug, i.e., 5-FU, induces cardiac complications, manifesting as ST-segment elevation and extended QRS duration [279]. Studies show that combining colchicine helps mitigate cardiac dysfunction by enhancing heart antioxidant capacity and reducing oxidative stress in heart cells [280].

Research has identified several other protective compounds against chemotherapy-induced cardiotoxicity, including quercetin, silymarin, and tanshinone IIA [281–283]. While curcumin has shown potential in preventing doxorubicin-induced cardiac cell death, these findings remain debatable [284].

Three compounds - curcumin, thymoquinone, and As_2_O_3_ - effectively reduce cisplatin-induced kidney damage by decreasing NF-κB and KIM-1 signaling, targeting Hh signaling, renal fibrosis, tubular injury, fibrosis severity, and ameliorating Nrf2/HO-1 signaling [285, 286]. However, clinical studies reveal concerning side effects of As_2_O_3_ in treating recurrent/resistant acute promyelocytic leukemia and multiple myeloma, including elevated serum creatinine, blood urea nitrogen, and proteinuria [287]. Consequently, further investigation is needed to determine if As_2_O_3_ is suitable as a nephroprotective agent during chemotherapy.

Resveratrol, a well-known natural antioxidant used for cardiovascular health and anti-aging, shows nephroprotective effects during chemotherapy. Experimental studies with mice receiving cisplatin treatment showed that resveratrol enhanced glomerular filtration rates by boosting SIRT1 levels, which subsequently reduced cisplatin-triggered p53 acetylation and cell death pathways [288].

[10]-Gingerol*(Zingiber officinale*), also exhibits multiple beneficial properties including antitumor effects, such as in breast cancer [289]. While combining [10]-Gingerol with doxorubicin didn’t significantly alter tumor size compared to doxorubicin alone in animal studies at 28 days, this combination therapy showed benefits in reducing chemotherapy-related weight loss and liver toxicity [290].

**Fig. 6:**
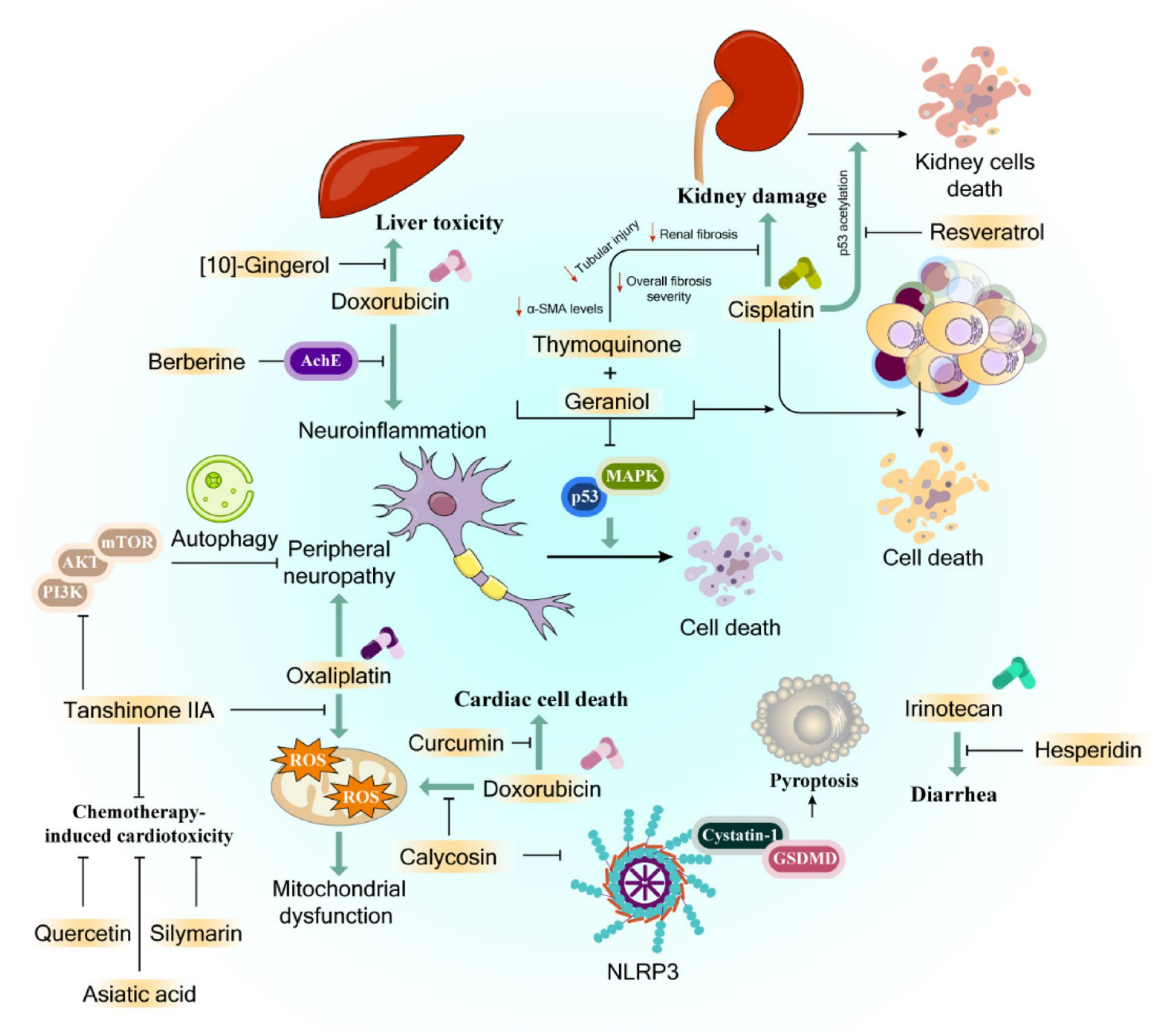
Signaling Mechanisms by which Natural Bioactives Reduce Side Effects of Chemotherapy. Key chemotherapeutic agents (doxorubicin, cisplatin, oxaliplatin, irinotecan) and their toxic effects, including hepatotoxicity, nephrotoxicity, neuroinflammation, peripheral neuropathy, mitochondrial dysfunction, cardiotoxicity, and diarrhea, are illustrated. Natural compounds including [10]-gingerol, berberine, thymoquinone, geraniol, resveratrol, tanshinone IIA, quercetin, silymarin, asiatic acid, curcumin, calycosin, and hesperidin mitigate these adverse effects through multiple mechanisms, such as inhibition of apoptosis and cell death pathways, modulation of autophagy, reduction of oxidative stress, suppression of MAPK and p53 pathways, and attenuation of neuroinflammatory responses. The mechanistic pathways highlighted include effects on ROS generation, NLRP3 inflammasome activation, mitochondrial function, and PI3K/AKT/mTOR signaling. Collectively, bioactive natural products are therapeutically potential as adjuvants in reducing chemotherapy-induced toxicity and improving patient outcomes. The figure was created in Adobe Illustrator

The therapeutic strategy of combining natural compounds with chemotherapy can enhance tumor-killing effects, reduce drug resistance development, and alleviate serious side effects, improving treatment outcomes (Fig. 6). Natural compounds, especially from traditional Chinese medicine, have been used for millennia in human diseases, providing active products and references for clinical research on combination chemotherapy. A natural compound often has multiple pharmacological activities, for example, tanshinone IIA can not only attenuate oxaliplatin-induced neurotoxicity, but also inhibit doxorubicin-induced cardiotoxicity, hepatotoxicity, and nephrotoxicity. Natural compounds in combination with chemotherapeutic agents are often more effective in their antitumor effects for reasons that are often not singular, and their beneficial results may be due to a combination of multiple mechanisms. Such multiple effects further confirm the feasibility of combining natural compounds with chemotherapeutic agents in the treatment of cancer. We can see that natural compounds have considerable potential to deal with the adverse reactions caused by chemotherapy, which can effectively alleviate the toxic effects caused by chemotherapy, assist the follow-up treatment of patients, and improve the quality of life.

## Natural bioactives in combination with chemotherapeutic agents: evidence from preclinical and clinical studies

The development of strategies that overcome cancer treatment resistance has become a considerable challenge; fortunately, natural products with diverse chemical structures and pharmacological effects have shown efficacy against drug resistance in cancer therapy. Here we review how natural compounds including polyphenols, alkaloids and terpenoids potentiate biological effects and reverse chemotherapy resistance in in vitro and in vivo cancer models.

### 5-Fluorouracil

Current research demonstrates curcumin’s ability to enhance chemotherapy and radiotherapy efficacy across cancer types [291]. Studies have shown that combining curcumin with 5-FU improves the response of treatment-resistant colon cancer cells, enabling reduced drug dosages [292]. Research incorporating 5-FU and curcumin demonstrated marked reduction in gastric cancer cell growth [293]. Shakibaei et al. investigated curcumin/5-FU combination effects on both wild-type HCT116 colon cancer cells and their 5-FU-resistant variants (HCT116R). Their findings revealed increased cell death, alongside decreased cell multiplication and colony development [7]. Gastric cancer studies yielded comparable results, with the curcumin/5-FU combination leading to heightened cell toxicity and decreased expression of COX-2 and NF-κB proteins. Furthermore, experiments with nude mice implanted with MKN45 cells showed greater tumor reduction when treated with both compounds compared to 5-FU alone [293].

Resveratrol (3,5,4’-trihydroxystilbene), a naturally occurring non-flavonoid polyphenol, is found in plants from *Vitis* and *Vaccinium* genera, including peanuts and grapes. It functions as a protective agent against UV radiation, oxidative stress, and fungal pathogens. Research has demonstrated resveratrol’s anti-inflammatory and antitumoral properties, as well as chemotherapy synergistic effects across cancer types, such as colorectal cancer [294–296]. Studies have revealed that resveratrol enhances cancer cells’ sensitivity to chemotherapeutic agents, promoting cell death and inhibiting inflammatory signaling cascades.

Buhrmann et al. investigated resveratrol’s effects alone and combined with 5-FU using three-dimensional models of HCT116 colon cancer cells and their 5-FU-resistant variants (HCT116R). Their experiment incorporated an inflammatory environment created by TNF-β and TNF-α cytokines. The findings revealed that resveratrol combined with 5-FU more effectively reduced the invasive capabilities of both cell types, even in the presence of TNF-β. This combination also regulated the NF-κB inflammatory pathway, increasing the cells’ responsiveness to 5-FU treatment [6].

Research has also established resveratrol’s capacity to suppress blood vessel formation in human breast cancer xenografts [297]. This compound affects the expression of HIF-1α and VEGF through multiple mechanisms, including PKB inhibition, MAPK activation, and suppression of blood vessel formation in ovarian cancer cells [298].

Lee et al. examined the anti-angiogenic effects of resveratrol and 5-FU individually and combined in a mouse melanoma model. Their research showed that combined treatment led to decreased microvessel density compared to control groups, suggesting effective angiogenesis inhibition. The combination therapy also resulted in reduced tumor growth and size, which was attributed to altered expression patterns of AMPK, VASP, and VEGF [299].

### Cisplatin

Studies have revealed that natural compounds can also synergize chemodrugs effects or reverse the existing resistance against them. In this context, oridonin is one these natural bioactives that has shown promise in sensitizing cisplatin-induced apoptosis via AMPK/Akt/mTOR-dependent autophagosome accumulation in A549 cells [300].

Furthermore, recent studies in colon cancer and lung adenocarcinoma models demonstrate that neferine combined with cisplatin increases intracellular cisplatin concentration. This combination intensifies both apoptotic and autophagic processes through ROS-mediated mechanisms, affecting MAPK and PI3K/Akt/mTOR pathways, while also activating the independent, non-canonical Beclin-1 and PI3K CIII pathway [301, 302].

In A549 lung cancer cells, the combination of resveratrol and cisplatin promotes autophagy by downregulating the PI3K/Akt/mTOR pathway. Additionally, this combination induces apoptosis through alterations in Bax and Bcl2 expression levels [303].

Kim et al. investigated how cisplatin combined with a polyphenol mixture DP-23 (E)-3-(3.5-d)-dimethoxyphenyl 1-(2-methoxyphenyl) prop-2-in-1) in cisplatin-resistant and -sensitive head and neck cancer cells. Their findings demonstrated enhanced ROS generation and Nrf2 suppression, leading to cancer cell death, including resistant strains, while sparing normal cells. This selective action is particularly significant since Nrf2 signaling typically promotes GST and GSR expression, enzymes known to contribute to cisplatin resistance [304].

Sirota et al. examined caffeic acid (a common dietary polyphenol) combined with cisplatin in ovarian cancer cells. Their research was motivated by caffeic acid’s known inhibitory effects on GST and GSR. The findings revealed that the combination enhanced cisplatin-induced cytotoxicity and increased platinum-DNA binding, indicating caffeic acid’s role as a sensitizing agent [305].

Cucurbitacin B, a tetracyclic triterpenoid from *Cucurbitaceae*, has attracted recent attention for its anticancer properties. When used alongside cisplatin, this compound enhanced toxic effects in both resistant and sensitive ovarian cancer cells. The mechanism involved increased ROS production, modulation of STAT-3 and ERK1/2 pathways, and decreased Dyrk1B levels [306]. Furthermore, this combination therapy significantly impeded tumor growth by triggering both caspase-dependent and independent pathways, promoting both autophagy and apoptosis in bladder cancer cells [307].

The isoquinoline alkaloid berberine demonstrates therapeutic potential for multiple diseases [308]. Current research highlights berberine’s anticancer properties against multiple malignancies, as it synergistically sensitizes human liver cancer cells to sorafenib [309]. Studies comparing berberine and cisplatin monotherapies versus combination therapy revealed enhanced growth inhibition in ovarian cancer models. This synergy occurred through increased caspases-3/8, RIPK3, and MLKL expression/activation in OVCAR3 and patient-derived ovarian cancer cells [310]. Additionally, low-concentration berberine enhanced cisplatin sensitivity in breast cancer cells by upregulating caspases 3 and 9, downregulating Bcl-2 protein, augmenting cisplatin-induced DNA damage, and reducing cellular PCNA levels [311].

Other alkaloids (e.g. tetrandrine) show similar cisplatin synergy in triple negative breast cancer models, enhancing ROS production and activating caspases [312]. In lung cancer cells, alkaloids such as dendrobine and sophoridine improved cisplatin chemosensitization. Notably, dendrobine demonstrated the additional benefit of reducing cisplatin-induced weight loss and cardiac toxicity in animal studies [313, 314].

Curcumin-cisplatin combination therapy in bladder cancer using 253 J-Bv and T24 cell lines and animal models has revealed enhanced cancer cell migration inhibition, increased phosphorylated MEK levels, and regulated phosphorylated ERK1/2 protein levels. The combination therapy produced higher ROS-mediated and ERK phosphorylation-dependent apoptosis compared to control or monotherapy groups. Animal studies showed significant tumor volume reduction without affecting body weight, suggesting good treatment tolerability [315].

Resveratrol-cisplatin combination therapy enhanced apoptosis and cytotoxicity in hepatoma cells (C3A, SMCC7721) but not normal LO2 cells, via glutamine metabolism inhibition. Notably, the combination therapy showed selective action, sparing normal LO2 cells while maintaining effectiveness against cancer cells [316].

Research on luteolin, another polyphenol, combined with cisplatin in cisplatin-resistant ovarian cancer cells (CAOV3/DDP) demonstrated dose-dependent growth inhibition and enhanced antiproliferative effects. The combination therapy augmented Bcl-2 downregulation and significantly reduced cell migration and invasion capabilities in resistant cells [317].

EGCG, green tea’s predominant catechin, also enhances conventional cancer treatment efficacy and reversing drug resistance; EGCG-cisplatin combination effects have been found on Copper Transporter 1 (CTR1) expression in ovarian cancer models. CTR1’s significance stems from its role in regulating intracellular cisplatin concentrations, with increased expression potentially enhancing cisplatin sensitivity. EGCG both induces CTR1 expression (mRNA and protein levels) and prevents its degradation. The EGCG-cisplatin combination increased intracellular chemotherapeutic accumulation, enhanced treatment sensitivity in ovary cancer cells, and effectively reduced tumor growth in animal studies [8].

The team further investigated non-coding RNAs regulating CTR1 expression during cisplatin-EGCG treatment of lung cancer cells. Their findings demonstrated that EGCG stimulates both CTR1 and lncRNA NEAT1 expression while suppressing miR-98-5p microRNA, which are suggested to be positive and negative CTR1 regulators, respectively. The combination therapy effectively inhibited tumor growth in lung cancer animal models, decreased Ki-67 expression, and enhanced cisplatin uptake in lung cancer cells, confirming CTR1 upregulation as a key mechanism in cisplatin sensitization [318].

While platinum drugs primary induce DNA damage, cancer cells counteract this through enhanced DNA repair, leading to resistance; research efforts have focused on identifying molecules that can inhibit these repair mechanisms. Small molecules NSC143099 and NSC16168 were found to inhibit ERCC1-XPF heterodimer activity, a crucial endonuclease in mammalian DNA repair pathways. This inhibition enhanced cisplatin-induced cytotoxicity in cancer cells and reduced tumor growth in animal models [319].

Due to EGCG’s 90% structural similarity to NSC143099, researchers evaluated EGCG-cisplatin combination effects in human cancer models. The study revealed EGCG as a potent ERCC1-XPF activity inhibitor, resulting in increased cisplatin sensitivity in tumor cells, enhanced cell death, and reduced proliferation [320].

### Doxorubicin

Despite doxorubicin’s effectiveness against carcinomas, sarcomas, and hematologic malignancies, its efficacy in colorectal cancer remains limited. This limitation primarily stems from P-glycoprotein (P-gp) overexpression, an ADP-dependent pump that expels xenobiotics like doxorubicin from cells, leading to treatment resistance [321].

Li et al. explored *Poria cocos*-derived triterpenoids (pachymic and dehydrotumulosic acid, PT) for enhancing chemotherapy efficacy. Using a liposomal co-delivery system for doxorubicin and PT in breast cancer models, they demonstrated that PT administration increased doxorubicin sensitivity in resistant MCF cells and enhanced anti-tumor effects in mice with doxorubicin-resistant breast cancer tumors, attributing these improvements to P-gp expression and function modulation [322].

Khaleel et al. investigated resveratrol and didox (DID) combined with doxorubicin in colon cancer. Their research revealed increased doxorubicin retention within cells due to P-gp efflux inhibition by both compounds. The study also showed enhanced expression of apoptotic markers BAX and TP53, along with decreased anti-apoptotic BCL-XL expression in HCT116 and HT29 cells treated with resveratrol/doxorubicin or DID/doxorubicin combinations, indicating increased apoptotic activity [323].

Borneol-doxorubicin combination enhanced cellular doxorubicin uptake and ROS production in glioma models, activating p53/p21 pathways, causing G2/M arrest and reduced angiogenesis [324]. In prostate cancer models, combining the alkaloid piperlongumine with doxorubicin enhanced apoptosis through increased caspase 3 and PARP activation, with the involvement of carbonyl reductase 1 inhibition [325].

Wen et al. examined curcumin’s effects on doxorubicin resistance in MCF-7/doxorubicin and MDA-MB-231/doxorubicin breast cancer cells. While doxorubicin remains a primary chemotherapeutic agent for breast cancer, its long-term effectiveness is compromised by resistance development. Their findings revealed that curcumin sensitized resistant cells to doxorubicin by inhibiting ABCB4 ATPase activity, an efflux carrier responsible for drug elimination, thereby enhancing intracellular drug accumulation and treatment efficacy [326].

*Xylopia vielana* leaf-derived terpenoids vielanin K show promise in combination with doxorubicin. Vielanin K enhanced doxorubicin sensitivity in resistant MCF-7 breast cancer cells through IRE1α-TRAF2-JNK pathway activation, triggering intrinsic apoptosis [327]. At subtoxic concentrations, vielanin P increased doxorubicin retention, reduced colony formation, and enhanced apoptosis by suppressing PI3K/Nrf2 pathway and MRP1 transporter function in resistant MCF-7 and K562 cells [328].

DOX’s utility is limited in osteosarcoma by dose-dependent cardiotoxicity (high doses) and reduced efficacy/drug resistance (low doses), thus necessitating alternative strategies; recent research has highlighted the role of long non-coding RNAs (lncRNAs) in tumor progression, particularly the SOX2 overlapping transcript (SOXOT) variants linked to cell differentiation and carcinogenesis [329]. Wang and colleagues demonstrated that EGCG-doxorubicin combination therapy decreased lncRNA SOXOT variant 7 expression in osteosarcoma cells, enhancing doxorubicin’s growth-inhibitory effects [330].

Namazi Sarvestani et al. examined eupatorine and salvigenin combined with doxorubicin in colon cancer cells (HT29, SW948) and normal fibroblasts (HFFF-2). The combination demonstrated synergistic effects, reducing cell viability while increasing ROS production and mitochondrial dysfunction, ultimately triggering mitochondrial-pathway apoptosis [331]. Similar synergistic outcomes have been documented when combining doxorubicin with various alkaloids and terpenes in cancers such as breast malignancies [332].

### Paclitaxel

Paclitaxel, a widely used taxane, is valued for its apoptosis-inducing properties in cancers [333]. Studies investigated its combination with naringin (4′,5,7-trihydroxyflavanone-aminoglucoside), a citrus-derived bioflavonoid known for its anti-inflammatory, antioxidant, and anticancer; Erdogan and colleagues in this context demonstrated that paclitaxel-naringin combination synergistically inhibited cell migration and enhanced PTEN tumor-suppressor protein expression in prostate cancer cells [334].

The protoberberine alkaloid coralyne, known for antileukemic activity, showed enhanced effects with paclitaxel in breast cancer cells. The combination produced greater reductions in ki-67 expression, decreased cancer cell-extracellular matrix interaction, increased Bax expression, and reduced Bcl levels compared to individual treatments, demonstrating synergistic antiproliferative and pro-apoptotic effects in MCF-7 and MDA-MB-231 cells [335].

*Nelumbo nucifera* and *Nymphaea caerulea*-derived nuciferine overcomes paclitaxel resistance by modulating PI3K/AKT/ERK pathway and suppressing Nrf2/HIF-1α activation and downstream targets (P-gp, BCRP) in resistant in-vitro and in-vivo cancer models. This led to increased intracellular chemotherapeutic accumulation, enhancing cytotoxicity and tumor suppression [336].

Additional alkaloids, including piperine and piperlongumine, showed promise in enhancing paclitaxel efficacy by increasing intracellular ROS in ovarian cancer SKOV-3 cells and intestinal cancer cells (INT-407 and HCT-116), respectively, promoting intrinsic apoptosis [337, 338].

### Other chemo-drugs

As a targeted therapy, imatinib (a tyrosine kinase inhibitor) inhibits several kinase proteins, specifically targeting the BCR-ABL gene fusion (formed by the Breakpoint Cluster Region and Abelson genes), platelet-derived growth factor receptors (PDGF-R), and c-kit [339]. Research has demonstrated that curcumin’s diverse therapeutic properties can enhance imatinib’s effectiveness and combat drug resistance in CML. This occurs through its ability to regulate the AKT/mTOR signaling pathway and decrease BCR/ABL gene expression, leading to cell death and growth inhibition [340].

A clinical case study by Demiray and colleagues documented the treatment of a 43-year-old patient with submandibular salivary duct adenoid cystic carcinoma. The patient, who had developed lung metastases and showed resistance to previous chemotherapy (cisplatin and etoposide combination), exhibited c-kit positive tumor cells. The treatment protocol combined daily imatinib (400 mg) with both intravenous curcumin (225 mg twice weekly) and oral curcumin supplement Arantal^®^ (84 mg twice daily). After 24 days, imaging revealed significant tumor reduction without mediastinal lymph node involvement. Follow-up at six months showed nearly complete anatomical and metabolic improvement, confirmed through physical examination and laboratory tests. Throughout the treatment period, no adverse effects were reported, and the intravenous curcumin administration proved safe and well-tolerated [341].

Docetaxel, a second-generation taxane similar to paclitaxel, treats various malignancies. At the cellular level, this compound exhibits strong binding affinity to β-tubulin, which disrupts microtubule dynamics and interferes with cytoskeletal function during cell division. This interference triggers G2/M phase cell cycle arrest, ultimately leading to growth suppression and cellular death [342].

Research has shown that when docetaxel is used in conjunction with piperlongumine, it demonstrates potent anti-cancer effects. This combination works by downregulating multiple growth-promoting and anti-apoptotic markers in breast cancer cells, particularly through the suppression of Bcl-2 and survivin. The protein survivin, which is commonly expressed across various cancer types, plays a crucial role in chemotherapy resistance and is associated with increased tumor recurrence and decreased patient survival rates [343].

Temozolomide, a DNA-alkylating agent that induces guanine nucleotide methylation, stands as a crucial chemotherapeutic drug for treating glioblastoma and astrocytoma [344]. Liu et al. demonstrated borneol enhances temozolomide’s efficacy in U251 glioma cells. This enhancement occurs through increased ROS generation and activation of mitochondrial-mediated apoptosis, leading to elevated levels of caspases 3, 7, and 9, and pro-apoptotic proteins Bax and Bad, while suppressing anti-apoptotic proteins Bcl-2 and Bcl-XL [345].

While gemcitabine serves as the primary chemotherapy for advanced pancreatic cancer, patients frequently develop drug resistance [346]. Research conducted by Lou and colleagues demonstrated that ginkgolide B, a terpene compound, could enhance gemcitabine sensitivity in pancreatic cancer cells by regulating cell proliferation and apoptosis, as evidenced in both cell cultures and Capan-1 cell xenograft tumor models [220].

Oxaliplatin, commonly used in treating various gastrointestinal cancers, has shown promising results when combined with polyphenolic compounds, including ellagitannin metabolites, lignans, and isoflavones. Norden et al. showed oxaliplatin-urolithin A combination affects HCT116 colon cancer proliferation via p53-dependent mechanisms. This combination increases the expression of p21 and TIGAR (TP53-Induced Glycolysis And Apoptosis Regulator), leading to cell cycle regulation and reduced glycolytic activity in cancer cells, ultimately restricting tumor growth [347]. Table 3 summarizes the most common synergistic effects of natural products in overcoming chemotherapy resistance.

**Table 3:** Synergistic effects of natural products in overcoming chemotherapy resistance

Natural compound	Chemotherapeutic agent	Cancer type	Mechanism of action	References
Curcumin	5-FU	Colorectal cancer Gastric cancer	miRNA-induced suppression of EMT Down-regulation of COX-2 and NF-κB signaling pathways	[292, 293]
Resveratrol	5-FU	Colorectal cancer	Chemosensitization of TNF-β-induced survival of 5-FU treated cells Increase in apoptosis (role of caspae-6 and p53)	[6, 296]
Neferine	Cisplatin	Lung adenocarcinoma Colorectal cancer	Induction of ROS-mediated non-canonical autophagy Increase in intracellular uptake of cisplatin and induction of ROS-mediated apoptosis	[301, 302]
Oridonin	Cisplatin	Lung cancer (A549 cells)	Sensitization of cisplatin-induced apoptosis via AMPK/Akt/mTOR-dependent autophagosome accumulation in cancer cells	[300]
Cucurbitacin B	Cisplatin	Ovarian cancer Bladder cancer	Increase in ROS, Modulation of STAT-3 and ERK1/2 pathways, and Induction of cell death pathways	[306, 307]
Berberine	Cisplatin	Ovarian cancer Breast cancer	Induction of necroptosis and apoptosis Induction of DNA breaks and caspase-3-dependent apoptosis	[310, 311]
EGCG	Cisplatin	Lung cancer Ovary cancer	NEAT-1-mediated up-regulation of EGCG-induced CTR1 to enhance cisplatin sensitivity in lung cancer cells Overexpression of CTR1 in ovary cancer	[8, 318]
Pachymic Acid (*Poria cocos* extract)	Doxorubicin	Breast cancer	Modulation of P-gp expression and increase in doxorubicin sensitivity	[322]
Resveratrol and Didox	Doxorubicin	Colorectal cancer	Amelioration of P-gp activity, increases BAX and TP53, decreases BCL-XL	[323]
Piperlongumine	Doxorubicin	Prostate cancer	Carbonyl reductase 1 inhibition	[325]
Vielanin K	Doxorubicin	Breast cancer	Activation of IRE1α-TRAF2-JNK pathway, Induces in intrinsic apoptosis	[327]
Naringin	Paclitaxel	Prostate cancer	Inhibition of migration and increase in PTEN expression	[334]
Coralyne	Paclitaxel	Breast cancer	Reduction of Ki-67, increase in Bax, decrease in Bcl-2, induction of apoptosis	[335]
Curcumin	Imatinib	c-kit-positive metastatic Adenoid Cystic Carcinoma	Not defined	[341]
Piperlongumine	Docetaxel	Triple-negative breast cancer	Enhancing oral bioavailability and cytotoxicity of docetaxel, suppression of survivin, induction of apoptosis	[343]
Borneol	Temozolomide	Glioma	Triggering mitochondrial dysfunction and ROS-mediated oxidative damage	[345]
Ginkgolide B	Gemcitabine	Pancreatic cancer	Inhibition of PAFR/NF-кB pathway	[220]
Urolithin A	Oxaliplatin	Colorectal cancer	Modulates p53-dependent pathways, induces p21 and TIGAR expression	[347]

## Preclinical and clinical studies on bioactive-chemotherapy combinations

Laboratory and clinical studies have evaluated natural compound-chemotherapy combinations, with curcumin being the most extensively studied. However, curcumin’s poor oral bioavailability remains a significant limitation [348]. To address this challenge, scientists have developed various solutions, including encapsulation techniques, specialized formulations, nanoparticle delivery systems, and metabolism-modifying additives [349].

A phase II trial investigated phospholipid-complexed curcumin (2000 mg/day) plus gemcitabine in 44 advanced/metastatic pancreatic cancer patients. The treatment demonstrated minimal neurological and blood-related toxicities while achieving a 61.4% disease control rate. The study reported a median progression-free survival of 8.4 months and overall survival of 10.2 months, surpassing gemcitabine monotherapy’s 6.7-month survival rate [350], and matching the 8.5–10.7 month survival observed with combined albumin-bound paclitaxel and gemcitabine [351, 352].

A phase IIa study compared FOLFOX alone versus FOLFOX plus oral curcumin (2 g/day Curcumin C3 Complex: 80% curcumin, 20% related compounds) in 28 metastatic colorectal cancer patients. The curcumin-enhanced treatment (CUFOX) proved safe, with comparable adverse event rates and Global health scores between groups, while achieving significantly better overall survival. The researchers also detected various curcumin compounds and metabolites in patients’ plasma, confirming treatment adherence [353].

Saghatelyan et al. conducted a randomized, double-blind, placebo-controlled trial of weekly intravenous curcumin (300 mg) plus paclitaxel in 150 advanced breast cancer patients. The curcumin group-maintained quality of life while showing superior objective response rates compared to placebo, with benefits persisting three months post-treatment [354]. However, the study didn’t compare plasma curcumin levels between oral and intravenous administration methods.

## Potential benefits and risks

Cancer development, prevention, and treatment involve multiple complex factors. Recent studies have highlighted phlegm formation as one significant contributor to cancer development [355]. The crucial role of phlegm in cancer progression has become a focal point of contemporary research [356]. Traditional herbal medicines have demonstrated capabilities in phlegm elimination, with numerous studies documenting their effectiveness in removing phlegm and maintaining proper blood qi circulation [357].

When examining the relationship between herbal medicines and chemotherapy drugs, the liver emerges as the primary site of interaction, where both substances undergo metabolic processes [358]. Certain herbal medicines can enhance chemotherapy effectiveness by modulating the P450 system or phase-II metabolism. These botanical interventions influence drug metabolism similarly to other factors such as grapefruit consumption, alcohol intake, smoking, and lifestyle choices [359].

The activation or suppression of metabolic enzymes in both phase-I and phase-II systems play a crucial role in carcinogenesis. Phase-I enzymes can transform some compounds into carcinogens, while inhibiting these enzymes may prevent carcinogen formation. Similarly, phase-II metabolic processes are vital for eliminating foreign substances, including carcinogens, from the body. Research has identified numerous herbal medicines that can modify these enzyme systems responsible for carcinogen metabolism and elimination. Notably, natural compounds that inhibit the P450 system can prevent certain substances from becoming carcinogenic, thereby reducing cancer risk [360–362].

Natural compounds that enhance phase-II metabolism facilitate the elimination of carcinogens through kidney excretion by promoting binding processes. Several cruciferous vegetables, including cabbage, cauliflower, Brussels sprouts, mustard greens, and kale, demonstrate anticarcinogenic properties by boosting phase-II enzyme activity [363].

Research has documented various interactions between herbal supplements and chemotherapy drugs. For instance, *Ginkgo biloba* and ginseng interact with multiple chemotherapy agents (including cyclophosphamide, etoposide, teniposide, and various vinca alkaloids) through CYP2C19 and CYP3A4 inhibition. Garlic affects dacarbazine metabolism via CYP3A4 inhibition, while *G. biloba* and grape seeds interact with anticancer antibiotics, alkylating agents, and platinum complexes through free radical scavenging. Valerian impacts cyclophosphamide through CYP2C19 inhibition, and kava kava shows broad cytochrome P450 inhibition [364].

While numerous plants exhibit anticancer properties, their combination with chemotherapy can significantly impact treatment outcomes, producing both beneficial and potentially risky effects [365]. Interactions can occur even without deliberate herb consumption, as common foods like spices, condiments, fruit juices, teas, coffee, and vegetable oils can influence chemotherapy effectiveness [366–368].

## Challenges and future prospects

Despite compelling evidence from in vitro and preclinical studies, many natural compounds face significant challenges in clinical application due to poor bioavailability. Curcumin exemplifies this issue, as its low aqueous solubility coupled with rapid metabolism prevents it from achieving therapeutic levels in systemic circulation. To overcome these barriers, recent advances have focused on innovative delivery systems that improve absorption and efficacy. For instance, nanoparticles have been developed to enhance solubility, protect unstable compounds, and enable targeted delivery, while liposomes facilitate better cellular uptake and reduce systemic toxicity. Additionally, phytosomal complexes—where natural molecules are bound to phospholipids—further increase bioavailability. The use of adjuvants like piperine has also proven effective by inhibiting metabolic enzymes responsible for rapid clearance, as seen in combinations like curcumin with piperine. Expanding discussion on these novel delivery approaches is vital to guide future translational research and enhance clinical applicability.

Equally important is a thorough evaluation of the safety profiles and toxicity risks associated with long-term use of natural compounds. Certain agents, such as arsenic trioxide used in treating specific leukemias, carry known nephrotoxic and cardiotoxic risks, highlighting the necessity for stringent dose monitoring. Understanding dose-limiting toxicities, organ-specific adverse effects, contraindications in patients with impaired liver or kidney function, and the potential for cumulative toxicity, especially when combined with chemotherapy, is essential to avoid harm and optimize treatment outcomes.

A major obstacle in herbal medicine research and clinical use is variability in extraction methods, purity, and bioactive compound content, which significantly hampers reproducibility and comparability of results. Addressing this problem requires the widespread adoption of standardized protocols, such as those outlined in the WHO Good Agricultural and Collection Practices (GACP) (https://wkc.who.int/resources/publications/i/item/9241546271). Moreover, rigorous quality control and assurance comparable to pharmaceutical-grade standards are crucial to ensure product consistency and patient safety. These considerations should be strongly emphasized as fundamental guidelines for the integration of herbal products into clinical practice.

Clinical translation of herbal medicines in oncology remains limited, with most evidence drawn from small Phase I and II trials and a scarcity of large randomized Phase III studies necessary for definitive validation. To surmount these gaps, it is imperative to conduct well-designed Phase III clinical trials that focus on both efficacy and safety. Cost-effectiveness analyses comparing natural adjuvants to synthetic drugs, especially in resource-constrained settings, are also critical. Additionally, implementing biomarker-driven patient stratification—for example, selecting patients exhibiting P-glycoprotein (P-gp) overexpression who might benefit from agents like schisandrin B—can help tailor therapies and improve clinical outcomes.

Looking forward, priority areas for research include investigating epigenetic modulators such as juglone, which acts as a Pin1 inhibitor with potential to affect oncogenic pathways. Exploring how natural compounds influence the microbiome to enhance tolerance and effectiveness of chemotherapeutics, such as irinotecan, also represents a promising domain. Furthermore, the development of triple-combination therapies that integrate chemotherapy, natural bioactives, and immunotherapy could harness synergistic anticancer effects and revolutionize treatment paradigms.

Regarding the nano-delivery of natural bioactives, polymeric nanoparticles, solid lipid nanoparticles, nanostructured lipid carriers, and micelles can enhance dissolution, shield labile scaffolds from enzymatic degradation, and enable passive (EPR effect) or active targeting (ligand-decorated surfaces). Design levers include particle size, surface charge (near-neutral to slightly negative to reduce opsonization), PEGylation (stealth), and ligand functionalization (e.g., folate, RGD, antibodies) for receptor-mediated uptake. Co-loading natural agents with chemotherapy (or with permeability/efflux modulators) can enable pharmacokinetic and pharmacodynamic synergy. Vesicular systems improve cellular uptake, modulate release kinetics, and can reduce systemic toxicity by altering biodistribution. Thermo- or pH-sensitive liposomes enable stimulus-responsive release in tumor microenvironments. Clinical precedence with liposomal chemotherapeutics provides a translational template for natural bioactives, including scalable manufacturing, stability, and quality-control paradigms. Conjugating polyphenols or terpenoids to phospholipids increases membrane permeability and lymphatic uptake, improving oral bioavailability without extensive chemical modification of the parent scaffold. Self-emulsifying drug delivery systems (SEDDS/SMEDDS), mucoadhesive polymers (e.g., chitosan) to prolong gastrointestinal residence, enteric coatings to bypass gastric degradation, and permeability enhancers for paracellular/transcellular transport can substantially increase oral bioavailability. For GI-targeted effects (e.g., microbiome modulation), colon-targeted coatings and prebiotic co-formulations are promising [369].

Given the demonstrated anticancer properties of numerous plants and their complex interactions with chemotherapy agents, an integrative approach to oncology is essential. Establishing a comprehensive evaluation system for each patient that accounts for herb-drug-food interactions will enhance treatment safety and efficacy. Applying stringent quality control standards to herbal products, equivalent to those used for conventional chemotherapy drugs, is equally important. Through careful incorporation of beneficial herbal interactions into integrative oncology protocols, alongside vigilant monitoring to prevent adverse effects, clinicians can optimize outcomes and safely expand the therapeutic arsenal against cancer.

Given the significance of these interactions, a comprehensive evaluation system for cancer patients undergoing chemotherapy is essential. This system should identify beneficial herb-drug combinations and subject herbal products to the same rigorous quality control standards as conventional chemotherapy drugs. With documented positive effects on various cancers affecting kidneys, lungs, gastrointestinal tract, breast, and blood, beneficial interactions should be carefully identified and incorporated into integrative chemotherapy approaches.

## Conclusions

The integration of natural bioactive compounds with conventional chemotherapeutics offers a credible path to enhancing efficacy while attenuating drug resistance, systemic toxicity, and treatment‑limiting adverse effects, with multimodal actions spanning NF-κB, PI3K/AKT/mTOR, NRF2/HO‑1, and related pathways and supported by combinations such as curcumin–5‑FU, resveratrol-cisplatin, and EGCG–doxorubicin. To realize clinical impact, however, preclinical gains must translate through rigorously designed trials that move beyond small, heterogeneous studies toward adequately powered, randomized evaluations with standardized endpoints and safety monitoring. Key barriers include poor and variable bioavailability, batch‑to‑batch variability of herbal sources, herb–drug interactions, and the CMC demands of complex formulations, alongside regulatory expectations for quality, consistency, and reproducibility. Priorities therefore include: employing advanced delivery platforms (e.g., lipid/polymeric nanoparticles, SEDDS, phytosome/phospholipid conjugates) with human‑relevant PK/PD characterization; adopting pharmacopeial-grade sourcing with GACP/GMP, analytical release specifications, and dose standardization; and aligning early clinical studies to scalable manufacturing and regulatory roadmaps. In conclusion, despite compelling preclinical synergy, high-quality randomized clinical trials are limited, and bioactive purity, PK variability, interaction risks, and regulatory barriers remain major hurdles to clinical adoption. With these translational constraints explicitly addressed, a multidisciplinary, precision‑oriented program can incorporate natural bioactives into oncology protocols to deliver more effective, safer, and patient‑centered therapies.

## Data Availability

No datasets were generated or analysed during the current study.
